# Cyclodextrin–Hydrogel Hybrids in Advanced Drug Delivery

**DOI:** 10.3390/gels11030177

**Published:** 2025-02-28

**Authors:** Hossein Omidian, Arnavaz Akhzarmehr, Erma J. Gill

**Affiliations:** Barry and Judy Silverman College of Pharmacy, Nova Southeastern University, Fort Lauderdale, FL 33328, USA; aa3853@mynsu.nova.edu (A.A.); eg1262@mynsu.nova.edu (E.J.G.)

**Keywords:** Cyclodextrin hydrogels, drug delivery systems, solubility enhancement, controlled release, biocompatibility

## Abstract

Cyclodextrin (CD)–hydrogel hybrids have emerged as versatile and multifunctional drug delivery systems, offering enhanced solubility, controlled drug release, and improved bioavailability. By combining the inclusion complexation properties of CDs with the swelling and retention capabilities of hydrogels, these hybrid systems overcome key challenges in conventional drug formulations. This review explores CD composition, hydrogel polymer selection, fabrication techniques, key drug release factors, and real-world therapeutic applications. Additionally, the latest advancements in stimuli-responsive hydrogels, nanogels, and microneedle-based drug delivery are discussed. While CD–hydrogel systems demonstrate significant potential, scalability, regulatory hurdles, and clinical translation remain key challenges. Future research should focus on smart hydrogels, improved drug loading strategies, and enhanced clinical validation to bridge the gap between laboratory innovations and commercial applications.

## 1. Introduction

The increasing demand for innovative drug delivery systems has propelled extensive research into hybrid materials that enhance drug solubility, bioavailability, and controlled release. Among these, cyclodextrin (CD)–hydrogel hybrids have emerged as a promising class of multifunctional drug carriers, capable of overcoming key limitations in conventional pharmaceutical formulations. CDs are cyclic oligosaccharides composed of α-(1,4)-linked glucopyranose units, forming a hydrophobic central cavity and a hydrophilic exterior. This unique structure enables them to encapsulate poorly water-soluble drugs through host–guest inclusion complexation, thereby improving drug solubility and stability [1,2,3,4]. Their pharmaceutical applications have expanded widely, but CD inclusion complexes alone often suffer from rapid drug release and insufficient mucoadhesion, necessitating further formulation advancements [5,6,7,8].

Hydrogels, three-dimensional hydrophilic polymer networks, have also gained prominence in drug delivery, regenerative medicine, tissue engineering, and biosensing. Their high water retention capacity allows for sustained drug release and excellent biocompatibility, making them ideal for ophthalmic, transdermal, mucosal, and injectable formulations [9,10,11,12]. However, conventional hydrogel systems often suffer from premature drug diffusion, inadequate mechanical stability, and limited site-specific targeting, which can compromise therapeutic efficacy [13,14,15,16]. To overcome these challenges, the incorporation of CDs into hydrogel networks has been explored to enhance drug loading capacity, controlled release, and formulation stability [17,18,19,20,21].

The synergy between CDs and hydrogels has led to the development of CD–hydrogel hybrid systems, combining the inclusion complexation ability of CDs with the swelling, retention, and sustained release properties of hydrogels. These hybrids have shown remarkable potential for multiple drug delivery routes, including ophthalmic, transdermal, oral, nasal, vaginal, rectal, and injectable formulations [6,18,22,23,24,25,26]. Furthermore, stimuli-responsive CD–hydrogel formulations—such as pH-sensitive, thermosensitive, and enzyme-responsive systems—have been designed to enable site-specific and on-demand drug release, improving therapeutic outcomes and patient compliance [27,28,29,30].

Despite their significant potential, CD–hydrogel hybrids face several challenges in clinical translation, including batch-to-batch variability, scalability limitations, regulatory constraints, and complex manufacturing processes [31,32,33,34]. Additionally, concerns related to stability, prolonged drug retention, and potential toxicity must be addressed before these systems achieve widespread adoption in pharmaceutical applications [35,36,37,38].

This review provides a comprehensive analysis of CD–hydrogel hybrids, focusing on formulation strategies, drug release mechanisms, and recent advancements in their application for advanced drug delivery systems. Key areas covered include CD composition, hydrogel polymer selection, fabrication techniques, factors influencing drug release performance, in vitro and in vivo evaluation methods, and therapeutic applications across various medical fields. Additionally, the review highlights current challenges and future directions, emphasizing the need for smart hydrogels, improved drug loading strategies, and enhanced clinical translation to fully unlock the potential of CD–hydrogel hybrid drug delivery systems.

## 2. Cyclodextrin Composition and Functionalization

CDs have emerged as essential excipients in modern drug delivery systems, due to their unique ability to form inclusion complexes with hydrophobic drugs. These cyclic oligosaccharides, composed of α-(1,4)-linked glucopyranose units, feature a hydrophilic exterior and a hydrophobic internal cavity, making them ideal for encapsulating poorly soluble drugs and enhancing their bioavailability. The three naturally occurring CDs—alpha-cyclodextrin (α-CD), beta-cyclodextrin (β-CD), and gamma-cyclodextrin (γ-CD)—differ in their cavity sizes and drug-binding affinities, enabling selective drug complexation based on molecular fit. Over the years, several chemically modified CDs, such as hydroxypropyl-beta-cyclodextrin (HP-β-CD), sulfobutyl ether- β-CD (SBE-β-CD), and randomly methylated β-CD (RAMEB or RM-β-CD), have been developed to further improve aqueous solubility, drug loading capacity, and controlled drug release [1,39].

### 2.1. β-CD and Its Derivatives: Improving Drug Solubility and Controlled Release

β-CD is one of the most widely utilized CD derivatives, due to its balanced hydrophobicity, biocompatibility, and ability to enhance drug solubility and stability. β-CD-based inclusion complexes have been extensively studied for topical, transdermal, vaginal, and oral formulations, particularly for drugs with low aqueous solubility. In analgesic and anti-inflammatory applications, β-CD inclusion complexes significantly improved lornoxicam solubility and dissolution, optimizing pain relief formulations [1]. Similarly, epichlorohydrin crosslinked β-CD (EPI-β-CD) nanogels provided sustained ibuprofen release, increasing dermal drug penetration by 3.5-fold, making them superior to conventional anti-inflammatory gels [5].

Beyond pain relief, β-CD has played a crucial role in antifungal drug delivery. Mucoadhesive β-CD-based vaginal gels demonstrated enhanced clotrimazole retention, prolonging antifungal activity for vaginitis treatment [2]. Additionally, β-CD nanosponges (β-CDNS), crosslinked using N,N-carbonyldiimidazole, significantly improved econazole nitrate permeation, leading to enhanced transdermal antifungal efficacy [16]. These findings highlight the ability of β-CD to prolong drug retention at targeted sites, while minimizing systemic exposure.

Furthermore, β-CD derivatives have been integrated into advanced hydrogel and nanogel systems for dermal and transdermal drug delivery. β-CD functionalized with hyperbranched polyglycerol in thermoresponsive nanogels significantly increased dermal drug penetration, optimizing topical drug diffusion and retention [40]. In dermatological applications, β-CD-decorated thermoresponsive nanogels for dexamethasone delivery demonstrated 30× higher dermal penetration, enhancing anti-inflammatory effects in skin-related disorders [41]. Another innovative application is the use of β-CD inclusion complexes in hydrogel-forming microneedles (HFMNs), which enabled sustained transdermal sildenafil citrate absorption, leading to improved bioavailability for erectile dysfunction therapy [42].

### 2.2. HP-β-CD: A Versatile Drug Solubilizer and Delivery Enhancer

HP-β-CD is a highly water-soluble β-CD derivative that is widely used in ophthalmic, transdermal, and oral drug formulations. One of its major advantages is minimal toxicity, making it suitable for sustained drug release and bioavailability enhancement. HP-β-CD has been particularly effective in antifungal therapy, where HP-β-CD-voriconazole vaginal gels significantly increased drug absorption into vaginal tissues, improving localized antifungal efficacy [6]. In ocular drug delivery, HP-β-CD-fluconazole ophthalmic gels provided controlled drug release and strong mucoadhesion, resulting in prolonged antifungal activity against ocular infections [7]. Similarly, HP-β-CD-ketoconazole inclusion complexes in ion-sensitive hydrogels enhanced ocular bioavailability by 47-fold, ensuring effective antifungal treatment with reduced dosing frequency [3].

Beyond antifungal applications, HP-β-CD has played a vital role in antibiotic therapy. HP-β-CD-azithromycin inclusion complexes in thermosensitive periodontal gels enabled sustained antibiotic release for over 54 h, optimizing localized periodontal disease treatment [43]. Another promising application is in oncology, where HP-β-CD-curcumin in situ gels significantly enhanced curcumin’s cytotoxicity against melanoma cells, showcasing HP-β-CD’s potential in cancer treatment [44].

In addition to gels and nanogels, HP-β-CD has been incorporated into microneedle-based and implantable drug delivery systems. HP-β-CD-risperidone inclusion complexes in hydrogel-forming microneedle array patches (HFMAPs) exhibited 4.75-fold increased drug solubility, allowing sustained transdermal delivery for up to 10 days—an alternative to intramuscular injections for schizophrenia treatment [45]. Moreover, HP-β-CD has been successfully polymerized onto hydroxyapatite bone implants, ensuring sustained antibiotic delivery, which is critical for bone infection management [46]. Similarly, HP-β-CD inclusion complexes with minoxidil in alginate-based hydrogels demonstrated enhanced scalp penetration and sustained release, optimizing alopecia treatment [47].

### 2.3. γ-CD: Expanding the Scope of Drug Complexation for Larger Molecules

γ-CD possesses a larger cavity size than β-CD, allowing it to accommodate bulkier and highly lipophilic drug molecules. This makes γ-CD particularly valuable in ophthalmic, transdermal, and antiviral drug formulations. For example, γ-CD nanogels crosslinked with HP-γ-CD significantly prolonged dexamethasone retention in ocular therapy, enhancing anti-inflammatory efficacy in ophthalmic treatments [48]. Furthermore, γ-CD-based metal–organic frameworks (γ-CD-MOFs) increased curcumin solubility by 267-fold, vastly enhancing bioavailability for anticancer therapy [17].

Another key application of γ-CD is in antiviral therapy. Valacyclovir-γ-CD inclusion complexes in topical gels improved dermal drug absorption, ensuring enhanced therapeutic efficacy against herpes simplex virus (HSV) [34]. Additionally, γ-CD crosslinked hydrogels for sustained josamycin release significantly increased drug loading efficiency, making them effective ocular implants for glaucoma treatment [49].

### 2.4. Functionalized Cyclodextrins: Next-Generation Drug Delivery Systems

Beyond conventional CD derivatives, functionalized CDs have been developed for specialized pharmaceutical applications. Sulfhydryl-beta-cyclodextrin (SH-β-CD) nanogels demonstrated mucoadhesive properties, enhancing fluconazole retention for oral thrush treatment [50]. Similarly, carboxymethyl-beta-cyclodextrin (CM-β-CD) physically crosslinked with chitosan improved antibacterial activity, making it ideal for antimicrobial hydrogel formulations [51].

In ophthalmic drug delivery, mono-methacrylated beta-cyclodextrin (MA-β-CD) crosslinked with polyvinyl alcohol (PVA) hydrogels reduced burst drug release, ensuring controlled ocular drug delivery in contact lens materials [37]. Additionally, Captisol, a chemically modified CD, has been successfully formulated into topical emulgels, significantly improving calcipotriol solubility and reducing adverse effects in psoriasis treatment [52].

Table 1 presents different types of CDs and their pharmaceutical applications, highlighting their key features, formulation characteristics, and reference numbers. β-CD is the most widely used, improving drug solubility and stability, particularly in oral and transdermal gels. HP-β-CD has higher aqueous solubility than β-CD, making it suitable for mucoadhesive and injectable hydrogels. γ-CD has a larger cavity, allowing it to encapsulate bigger molecules, benefiting ophthalmic and sustained-release formulations. CD nanosponges (CDNSs) provide enhanced drug stability and encapsulation efficiency. SBECD is highly soluble and often used in systemic drug delivery. CM-β-CD enables pH-responsive drug release, while Mβ-CD improves membrane permeability for nasal and dermal drug applications.

## 3. Hydrogel Polymers and Their Role in CD–Hydrogel Drug Delivery

Hydrogels are three-dimensional, hydrophilic polymer networks that can absorb and retain large amounts of water, making them ideal for controlled and sustained drug delivery. These systems are highly adaptable and can be tailored to respond to environmental stimuli such as pH, temperature, and ionic strength, ensuring precise drug release. When combined with CDs, hydrogels enhance drug solubility, bioavailability, and retention, making them suitable for ophthalmic, transdermal, mucosal, and bone regeneration therapies. Various hydrogel formulations, including thermosensitive, pH-responsive, mucoadhesive, and crosslinked systems, have been explored to optimize drug loading, release kinetics, and therapeutic efficacy.

### 3.1. Thermosensitive Hydrogels: Temperature-Responsive Drug Release Systems

Thermosensitive hydrogels undergo sol–gel transitions in response to temperature changes, making them suitable for localized and controlled drug delivery. Poloxamer-based in situ gels have been widely used for CD–drug inclusion complexes, allowing temperature-dependent sol–gel transitions for periodontal and ophthalmic applications. For example, HP-β-CD-azithromycin inclusion complexes in poloxamer-based hydrogels provided sustained antibiotic release, significantly improving periodontal therapy outcomes [43].

In cancer therapy, thermoresponsive poloxamer hydrogels have been utilized for curcumin-HP-β-CD complexes, ensuring sustained transdermal delivery and improved anticancer efficacy for melanoma treatment [44]. Additionally, HP-β-CD-dexamethasone thermoresponsive in situ gels, incorporating zinc-hyaluronate (ZnHA) and hydroxypropyl methylcellulose (HPMC), provided improved ocular drug retention and bioavailability, making them effective for ophthalmic corticosteroid therapy [72]. Another promising application is Pluronic F127-poly(ethylene oxide) (PEO) hydrogels, which facilitated temperature-responsive buccal delivery of paclitaxel, optimizing anticancer drug absorption and retention [71].

### 3.2. pH-Responsive Hydrogels: Targeted Drug Release Based on pH Sensitivity

pH-responsive hydrogels modulate drug release based on environmental pH changes, ensuring site-specific drug release in ocular, oral, and gastrointestinal therapies. HP-β-CD-ketoconazole inclusion complexes in ion-sensitive hydrogels significantly enhanced ocular antifungal drug delivery, achieving a 47-fold increase in bioavailability and prolonged drug retention in the cornea [3]. Similarly, HP-β-CD-based ophthalmic gels containing ciprofloxacin exhibited pH-induced gelation, optimizing drug residence time in the ocular cavity and improving antibacterial efficacy [22].

For oral and gastrointestinal drug delivery, pH-responsive HP-β-CD-grafted poly(methacrylic acid) (PMAA) hydrogels provided controlled cytarabine release, effectively prolonging plasma half-life for colon cancer therapy [56]. Similarly, sodium alginate-grafted hydrogels incorporating HP-β-CD-Puerarin inclusion complexes demonstrated pH-sensitive drug release, ensuring maximized bioavailability in acidic and neutral environments [73]. Another β-CD-grafted hydrogel, incorporating montmorillonite (MMT) nanocomposites, was formulated for lovastatin delivery in hyperlipidemia management, ensuring sustained release and improved therapeutic outcomes [14].

### 3.3. Mucoadhesive Hydrogels: Prolonging Drug Retention and Absorption

Mucoadhesive hydrogels enhance drug retention on mucosal surfaces, providing sustained therapeutic effects for wound healing, vaginal, and ophthalmic drug delivery. Carbopol-based β-CD and HP-β-CD hydrogels have been formulated to enhance allantoin’s wound-healing properties, offering strong antimicrobial activity against Staphylococcus aureus and Candida albicans [13]. In vaginal drug delivery, Poloxamer 407 and Poloxamer 188/HP-β-CD-based mucoadhesive gels improved voriconazole uptake, enhancing localized antifungal therapy [6].

Mucoadhesive polymeric nanogels, such as SH-β-CD-carbopol/gelatin systems, demonstrated improved oral drug retention, increasing fluconazole’s efficacy for oral thrush treatment [50]. Similarly, chitosan-based hydrogels, such as CS/CMCD physically crosslinked hydrogels, exhibited sustained drug release, while maintaining strong antimicrobial effects against E. coli, S. aureus, and C. albicans, making them effective for infection management [51].

### 3.4. Hydrogels for Ophthalmic and Nasal Drug Delivery

Hydrogels have been extensively used in ocular drug delivery systems to improve corneal permeability and sustained drug retention. HEMA/NVP-based hydrogel matrices, combined with chitosan-functionalized beta-CD, optimized drug permeability and protein resistance, ensuring long-term ophthalmic therapy [29]. Similarly, polyvinyl alcohol (PVA) hydrogels crosslinked with MA-β-CD reduced burst drug release, sustaining therapeutic concentrations of ocular drugs for 15 days [37]. Additionally, γ-CD crosslinked hydrogels effectively sustained josamycin release, offering long-term therapy for glaucoma treatment [49].

For nasal drug delivery, M-β-CD nasal inserts, combined with carrageenan and PVP90, provided prolonged estradiol release, ensuring steady hormone absorption for hormone replacement therapy [33]. Similarly, poloxamer 407 and PVP K30 hydrogels, loaded with asenapine maleate, significantly enhanced bioavailability and absorption via nasal administration, making them an effective alternative to oral schizophrenia treatments [28].

### 3.5. Microneedle Hydrogel Systems and Bone Regeneration Hydrogels

Microneedle-based hydrogel systems have gained significant attention for transdermal drug delivery. β-CD-based hydrogel-forming microneedles (HFMNs) optimized sildenafil transdermal bioavailability, outperforming oral Viagra^®^ formulations in terms of drug retention and systemic absorption [42]. Similarly, hydrogel-forming microneedles (HFMNs) with HP-β-CD inclusion complex reservoirs enabled sustained risperidone delivery, offering a long-term schizophrenia treatment alternative to intramuscular injections [45].

For bone regeneration applications, HP-β-CD-functionalized hydroxyapatite bone implants demonstrated controlled antibiotic release, significantly reducing infection risks during bone repair [46]. This approach offers sustained local drug delivery, preventing systemic side effects while enhancing bone healing.

### 3.6. Solvent-Exchange-Induced In Situ Gels (ISGs) and Microparticles (ISMs)

Solvent-exchange-induced hydrogels have been developed to extend drug release profiles. β-CD in solvent-exchange ISG/ISM systems successfully extended meloxicam release for up to 7 days, optimizing periodontal drug therapy [59]. These formulations ensure minimal burst release, providing consistent therapeutic action over extended periods.

Table 2 categorizes various hydrogel polymers based on their key features, applications, and formulation characteristics in drug delivery systems. Poloxamer 407 is widely used for thermosensitive hydrogels, transitioning from liquid to gel at body temperature, enhancing drug release. Carbopol is known for its high viscosity and mucoadhesion, making it suitable for controlled-release formulations in ophthalmic, transdermal, and vaginal gels. Hydroxypropyl methylcellulose (HPMC) is often combined with thermogelling agents to improve viscosity and bioadhesion. Chitosan forms pH-sensitive gels, and is commonly used in nasal, ocular, and oral drug delivery. Polyvinyl alcohol (PVA) provides mechanical strength and biocompatibility for ophthalmic and transdermal applications. Gelatin is biodegradable and frequently used in injectable and microneedle applications. CD-based nanosponges extend drug stability and release through crosslinked networks. Poly(ethylene glycol) (PEG) is often chemically crosslinked to enhance stability and solubility. Hydroxyethyl Methacrylate (HEMA) is ideal for contact lens drug delivery, due to its high water content and oxygen permeability. Methylcellulose (MC) exhibits temperature-sensitive properties, forming thermogels at physiological temperatures. These polymers play critical roles in hydrogel formulations, enabling controlled drug release, mucoadhesion, and targeted delivery.

## 4. Preparation Methods for CD–Hydrogel Hybrids

The preparation of CD–hydrogel hybrid systems involves multiple key steps, including drug–CD inclusion complex formation, hydrogel synthesis, polymer crosslinking, and formulation optimization. Further fabrication techniques such as kneading, spray-drying, solvent evaporation, emulsification, photopolymerization, and ionic crosslinking have been developed to tailor drug release profiles, prolong retention, and improve drug stability. The selection of a preparation method depends on the physicochemical properties of the drug, delivery route, and therapeutic application [1,39].

### 4.1. CD Inclusion Complex Formation

The formation of drug–CD inclusion complexes is a critical step in improving drug solubility, stability, and bioavailability. Various techniques, including kneading, spray-drying, solvent evaporation, and co-lyophilization, are employed to obtain stable drug–CD complexes.

The kneading method was effectively used for lornoxicam-β-CD and HP-β-CD inclusion complexes, which ensured enhanced drug solubility and controlled release in liquid crystalline gels, improving anti-inflammatory efficacy [1]. Similarly, ion-sensitive in situ gelation was applied to HP-β-CD-ketoconazole complexes, leading to optimized ocular antifungal therapy with a 47-fold increase in bioavailability [3].

For antifungal applications, fluconazole-β-CD solid dispersions were obtained using solvent evaporation, significantly enhancing gel performance and transdermal drug diffusion [53]. In vaginal drug delivery, HP-β-CD-voriconazole complexes were prepared using spray-drying, increasing drug absorption into vaginal tissues, thereby enhancing antifungal efficacy [6]. HP-β-CD-dexamethasone inclusion complexes, optimized using phase solubility studies, achieved a solubility constant of 258.62 M^−1^, ensuring prolonged ocular anti-inflammatory effects [4].

In diabetes therapy, MCD-insulin inclusion complexes were encapsulated into PMAA-chitosan microparticles, resulting in enhanced oral insulin absorption and improved glucose regulation [30]. Similarly, CMCD-grafted chitosan hydrogels, synthesized via carbodiimide crosslinking, enabled pH-sensitive insulin release, ensuring sustained blood glucose control [67].

### 4.2. Hydrogel Synthesis and Crosslinking Techniques

Hydrogel synthesis plays a crucial role in controlling drug release rates, improving bioadhesion, and enhancing drug stability. Several polymerization and crosslinking methods, including free radical polymerization, photopolymerization, and ionic crosslinking, have been employed to develop stable hydrogel drug carriers.

Poloxamer-based thermosensitive gels were prepared using cold processing methods, which provided temperature-responsive sol–gel transitions, ensuring sustained ciprofloxacin release for ophthalmic therapy [22]. Carbopol-based hydrogels containing HP-β-CD-allantoin inclusion complexes were developed with mucoadhesive and self-healing properties, enhancing wound healing and antimicrobial efficacy [13].

For antibacterial applications, physically crosslinked chitosan-CD hydrogels were synthesized using ionic interactions, enabling prolonged drug retention and extended therapeutic effects [51]. In dermatological applications, photopolymerization was used to fabricate dextran-CD cryogels, ensuring stable dermal curcumin delivery for cutaneous T-cell lymphoma treatment [27]. Additionally, poloxamer-based hydrogels were optimized for sol–gel transitions, enabling intranasal applications for enhanced bioavailability [26,28].

For ophthalmic formulations, UV polymerization was applied to PVA hydrogels crosslinked with MA-β-CD, allowing sustained ocular drug release for up to 15 days [37]. Similarly, freeze-dried nasal inserts, incorporating estradiol-Mβ-CD complexes, were designed with optimized moisture uptake and bioadhesion properties, prolonging hormone replacement therapy effects [33].

### 4.3. Nanogel and Emulsification-Based Formulations

Emulsification and solvent evaporation techniques are widely used in the preparation of nanogels, enabling enhanced drug retention and targeted drug delivery.

For ophthalmic inflammation therapy, γ-CD nanogels crosslinked with HP-γ-CD were synthesized via emulsification/solvent evaporation, ensuring prolonged dexamethasone retention and improved corneal absorption [48]. Similarly, flurbiprofen-loaded CD nanogels, incorporated into HPMC hydrogels, facilitated sustained drug release for 48 h, optimizing anti-inflammatory ocular therapy [75].

In mucoadhesive gel formulations, β-CD mucoadhesive gels for clotrimazole were designed using Pluronic F127, Carbopol 934, and HPMC, ensuring sustained vaginal antifungal activity [2]. Additionally, β-CDNS-based econazole nitrate hydrogels were optimized via a Box–Behnken design, improving nanoparticle size and drug release efficiency [16].

For topical drug delivery, β-CD-decorated thermoresponsive nanogels were synthesized for targeted dexamethasone skin penetration, ensuring efficient epidermal absorption [41].

### 4.4. Microneedle Patch Fabrication

Microneedle patches have emerged as an innovative drug delivery platform, providing controlled transdermal drug absorption with minimal invasiveness. Specialized fabrication techniques, such as photopolymerization and crosslinking, are essential for ensuring mechanical integrity and sustained drug release.

For melanoma treatment, GelMA-β-CD microneedles were developed via photopolymerization, allowing stable transdermal curcumin delivery [77]. Similarly, hydrogel-forming microneedles (HFMNs) containing β-CD inclusion complexes were fabricated to enhance sildenafil transdermal bioavailability, outperforming oral formulations [42].

In schizophrenia therapy, HP-β-CD-HFMN patches, produced using PVA and PVP, demonstrated strong mechanical properties and sustained risperidone release, providing an alternative to intramuscular injections [45].

### 4.5. Advanced Crosslinked and Bone Implant Hydrogels

Crosslinked hydrogels have been designed to provide long-term drug release, site-specific delivery, and enhanced biocompatibility.

For periodontal therapy, solvent-exchange ISG/ISM systems incorporating β-CD were developed to extend meloxicam release up to 7 days, optimizing inflammation control [59]. Hydroxyapatite-CD hybrid bone implants, crosslinked using butanetetracarboxylic acid, enabled sustained ciprofloxacin and vancomycin release, reducing infection risks in orthopedic surgeries [46].

Table 3 outlines various processes and techniques used to incorporate CDs into drug formulations. The kneading method is widely used for drug–CD complexation, improving solubility and bioavailability with minimal solvent use. Solvent evaporation ensures stable inclusion complexes by dissolving the drug and CD in a common solvent, followed by controlled evaporation. Freeze-drying (lyophilization) enhances drug stability, especially for ophthalmic and injectable applications. Spray-drying produces fine drug–CD powders, improving dissolution and bioavailability. Nanogel formation crosslinks CDs with polymers to create highly swellable drug-loaded nanogels. Emulsification–solvent evaporation is applied in nano- and microgel formulations for sustained release. Crosslinking methods, both chemical and physical, generate stable hydrogel matrices. pH-induced gelation and thermoresponsive gelation enable environmentally responsive drug release. Lastly, microneedle array technology facilitates transdermal drug delivery through patches with controlled release properties. These techniques optimize drug stability, solubility, and bioavailability, ensuring targeted and efficient drug delivery across various applications.

## 5. Key Factors Influencing Drug Delivery Performance

The effectiveness of CD–hydrogel hybrids is governed by CD type, drug complexation ratio, hydrogel composition, crosslinking density, environmental sensitivity (pH, temperature, mucoadhesion), particle size, and delivery route. Optimizing these factors ensures precise drug delivery, prolonged therapeutic action, and reduced systemic toxicity.

### 5.1. Swelling and Drug Release Control

Swelling behavior plays a crucial role in drug solubility, loading capacity, and release kinetics. Hydrogels with increased swelling ratios allow for enhanced drug diffusion, particularly in pH-sensitive and transdermal formulations. For instance, HP-β-CD-g-MAA hydrogels swelled up to 44.56 at pH 7.4, improving cytarabine release through increased hydration and drug solubilization [56]. Similarly, β-CD porous hydrogel nanoparticles enhanced solubilization and retention of hydrophobic drugs, making them highly effective for improving drug bioavailability [8,18].

Swelling is also crucial in transdermal microneedle applications; GelMA-β-CD microneedles swelled post-insertion, forming microchannels that facilitated sustained curcumin delivery in melanoma treatment [77]. Additionally, β-CD-chitosan hydrogels optimized swelling at alkaline pH, allowing for controlled lovastatin release [14]. Similarly, CM-CD-grafted hydrogels facilitated pH-sensitive insulin delivery, ensuring stability in gastric conditions while enhancing intestinal uptake [67].

### 5.2. CD Type and Drug Complexation Ratio

The type of CD and the ratio in which it complexes with the drug significantly influence drug solubility, dissolution, and bioavailability. The binding efficiency between CD and the drug determines inclusion complex stability, which directly impacts release profiles and therapeutic efficacy. For instance, lornoxicam-β-CD inclusion complexes at a 1:2 ratio exhibited optimal dissolution, improving topical bioavailability and anti-inflammatory effects [1]. Likewise, HP-β-CD-fluconazole inclusion complexes enhanced ophthalmic retention, ensuring sustained antifungal therapy and reducing frequent dosing requirements [7]. The application of γ-CD metal–organic frameworks (γ-CD-MOFs) dramatically increased curcumin solubility by 267.1-fold, reinforcing their ability to encapsulate hydrophobic drugs and significantly enhance bioavailability [17].

### 5.3. Gelation Temperature and Thermosensitive Performance

Gelation temperature governs hydrogel usability, stability, and drug retention. Thermosensitive hydrogels transition from liquid to gel at physiological temperatures, ensuring prolonged retention in ocular, vaginal, nasal, and tumor therapy applications. For example, Pluronic F127 hydrogels gelled above 25 °C, forming stable intratumoral depots for sustained chemotherapy [24]. Similarly, Poloxamer 407-based HP-β-CD gels gelled at physiological temperatures, ensuring prolonged ocular, vaginal, and nasal drug release [6,19,22,28,63]. Thermoreversible rectal films also significantly improved 5-FU absorption, increasing cellular uptake by 5.4-fold, making them an ideal candidate for colorectal cancer therapy [25].

### 5.4. Hydrogel Matrix Composition and Crosslinking Density

The polymer composition and crosslinking density of hydrogels control drug retention, stability, and release rates. A higher crosslinking density leads to slower drug diffusion, ensuring sustained release, while a lower density results in faster drug permeation. In vaginal drug delivery, Carbopol 934-based mucoadhesive hydrogels prolonged clotrimazole release, improving antifungal efficacy [2]. Similarly, β-CD nanosponges (β-CD-NS) enhanced drug stability and transdermal absorption, achieving a 10-fold increase in curcumin release and a 2.5-fold increase for resveratrol, reinforcing their potential in cancer and antioxidant therapies [65]. In addition, β-CD-g-MAA hydrogels exhibited 93.16% drug release at higher pH, making them ideal for statin delivery in hyperlipidemia therapy [14].

### 5.5. Environmental Sensitivity: pH, Temperature, and Mucoadhesion

CD–hydrogel hybrids are often designed to respond to pH, temperature, and mucoadhesion, enabling site-specific and prolonged drug release. For example, HP-β-CD-ketoconazole ophthalmic gels exhibited pH-induced controlled drug release, leading to a 47-fold improvement in bioavailability, making them highly effective for ocular antifungal therapy [3]. Likewise, poloxamer-based thermosensitive gels enabled sustained ciprofloxacin release, improving ocular infection treatment by increasing drug retention at the site of infection [22]. Furthermore, Estradiol nasal inserts demonstrated controlled hormone release, achieving 49% bioavailability, ensuring efficient hormone replacement therapy while minimizing systemic side effects [33].

### 5.6. Particle Size and Drug Permeation Efficiency

**Figure 1:**
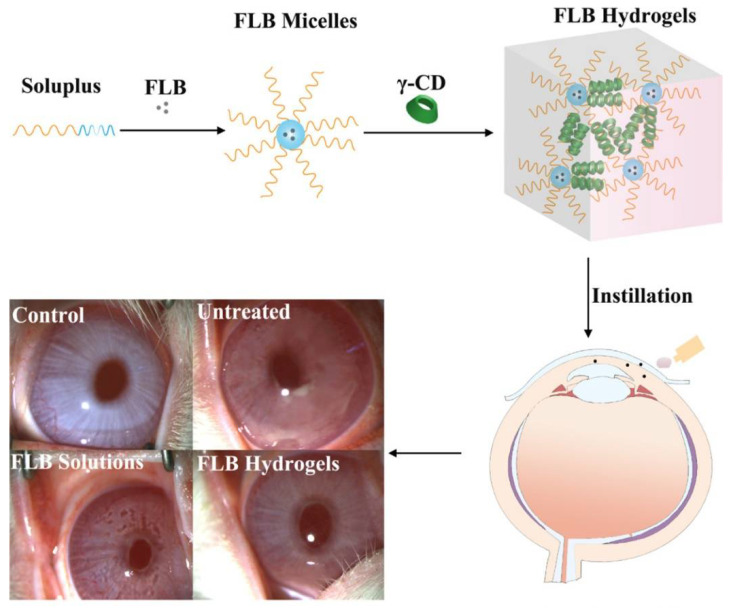
Illustration of preparation of γ-CD-based polypseudorotaxane hydrogels and topical delivery of FLB for treatment of anterior uveitis. Adopted with permission [64].

Particle size plays a critical role in drug permeability, diffusion rates, and overall bioavailability. Smaller particles enhance absorption and cellular uptake, whereas larger particles support sustained release and prolonged drug action. For instance, flurbiprofen-loaded γ-CD hydrogels increased corneal permeability by 1.84-fold and intraocular drug retention by 21.2-fold, making them highly effective for ophthalmic bioavailability. Polypseudorotaxane hydrogels enhanced transcorneal permeability, achieving prolonged flurbiprofen (FLB) release in rabbit models, in comparison with drug solutions and micelles, as shown in Figure 1. Polypseudorotaxane hydrogels were successfully prepared by the host–guest interaction between γ-CD and the PEG chains of Soluplus micelles. In addition, FLB hydrogels boosted anti-inflammatory efficacy in a rabbit model of endotoxin-induced uveitis at a reduced administration frequency [64]. (Figure 1).

Similarly, dexibuprofen-loaded β-CD nanoparticles significantly enhanced solubility and dissolution rates, supporting their application in oral and systemic drug delivery [8,18]. In dermatological treatments, β-CD-decorated nanogels achieved 30-fold higher dexamethasone dermal penetration, significantly enhancing localized anti-inflammatory effects [41].

### 5.7. Delivery Route and Bioavailability Optimization

The route of administration plays a major role in drug bioavailability and therapeutic impact. Different CD–hydrogel hybrids have been successfully employed in oral, transdermal, nasal, ophthalmic, and periodontal drug delivery systems. For instance, HP-β-CD-paliperidone nasal gels improved drug permeation, ensuring sustained and controlled release for schizophrenia therapy [74]. In ophthalmology, α-CD-poloxamer hydrogels significantly enhanced ciprofloxacin pre-corneal retention, ensuring prolonged antibacterial activity [70]. In periodontal therapy, β-CD-based ISG/ISM formulations extended meloxicam release for 7 days, significantly reducing inflammation in periodontitis treatment [59]. Similarly, in dermatological applications, HP-β-CD-minoxidil inclusion complexes increased solubility by 22-fold, making them effective for hair regrowth therapy in alopecia patients [47].

Table 4 highlights key factors influencing drug release in CD-based hydrogel systems, emphasizing their effects, involved CD types, and technical considerations. CD type and inclusion complex formation significantly impact drug solubility and bioavailability, with β-CD, HP-β-CD, and SBECD commonly used. Hydrogel polymer composition and crosslinking density determine drug diffusion rates, with higher crosslinking leading to slower release. pH sensitivity enables responsive drug release in oral and vaginal applications, while temperature-sensitive gelation triggers release at physiological temperatures. Swelling and degradation of the hydrogel matrix control sustained release over time. Drug hydrophobicity and molecular weight influence release rates, with smaller drugs diffusing faster. Ionic strength and osmotic effects impact mucoadhesive and ophthalmic formulations. Microneedle patch swelling enhances transdermal penetration, while emulsification and nanoparticle size improve diffusion for ocular and topical delivery. Iontophoresis and electrostimulation accelerate drug permeation in transdermal applications, especially for pain management and low-permeability drugs. These factors collectively optimize drug release profiles, ensuring controlled, site-specific, and effective drug delivery.

## 6. Evaluation and Characterization of CD–Hydrogel Systems

CD–hydrogel hybrid drug delivery systems have demonstrated significant potential in enhancing drug solubility, bioavailability, and controlled release. However, their effectiveness and safety must be rigorously evaluated using a combination of physicochemical, biological, and pharmacokinetic studies. This section outlines the critical real-world testing and assessment approaches required for CD–hydrogel formulations, ensuring comprehensive characterization and optimization.

### 6.1. Physicochemical Characterization and Material Analysis

A thorough understanding of the structural, thermal, and solubility properties of CD–hydrogel formulations is essential for ensuring stability, drug compatibility, and effective drug delivery.

- Structural and Spectroscopic Analysis: Fourier-transform infrared spectroscopy (FTIR), X-ray diffraction (XRD), nuclear magnetic resonance (^1^H NMR), and powder X-ray diffraction (PXRD) confirm the formation of drug–CD inclusion complexes, assess crystallinity, and evaluate molecular interactions within hydrogel matrices [1,2,4,14,18,34,50,67,68].
- Thermal Stability and Degradation Studies: Differential scanning calorimetry (DSC) and thermogravimetric analysis (TGA) determine the stability of CD–hydrogel formulations by analyzing phase transitions, drug–polymer interactions, and degradation under thermal stress [2,4,8,16,18,34,54,56].
- Surface Morphology and Porosity: Scanning electron microscopy (SEM) and transmission electron microscopy (TEM) are used to assess hydrogel porosity, homogeneity, and surface integrity, which influence drug loading, swelling, and release characteristics [8,14,34,58,68,79].
- Solubility and Hydration Properties: Phase solubility studies, equilibrium swelling ratio, and water uptake assessments help to evaluate how CD–hydrogels enhance drug solubility and hydration behavior, which is critical for effective drug release and bioavailability [1,2,4,5,17,34,50,63,76].

### 6.2. Rheological and Gelation Behavior

Hydrogels must demonstrate appropriate viscosity, swelling behavior, and gelation properties for optimal performance across different drug delivery applications.

- Gelation Behavior and Mechanical Strength: Gelation temperature, viscosity, and viscoelastic properties are assessed to ensure hydrogel stability and usability, particularly in injectable, mucosal, and transdermal formulations [2,6,21,28,63,69,75,76].
- Rheological and Swelling Studies: Shear viscosity, swelling ratio, and rheological properties are evaluated to optimize hydrogel consistency and ensure adequate drug diffusion and retention, particularly in ophthalmic and transdermal applications [14,20,23,24,37,43,56,60].

### 6.3. Drug Release and Permeation Studies

Efficient and sustained drug release, along with adequate membrane permeation, are essential for the success of CD–hydrogel drug delivery systems.

- In Vitro Drug Release and Kinetics: Controlled release profiles are studied using dissolution tests, mathematical release kinetics (e.g., Higuchi, Korsmeyer-Peppas models), and pH-dependent diffusion studies to optimize therapeutic performance [1,2,4,8,14,16,21,22,34,63,73,76,80].
- Ex Vivo and Transdermal Permeation Studies: Franz diffusion cells and other membrane permeability studies are used to assess drug penetration through skin, corneal tissue, and mucosal surfaces, optimizing formulations for ophthalmic, transdermal, and nasal delivery [1,3,17,34,47,61,65,70,76].
- Cellular Uptake and Transport: Permeability and absorption efficiency are analyzed using cellular models such as Caco-2 cells, nasal epithelial cell monolayers, and Ussing chambers, providing insights into systemic and targeted drug delivery potential [13,15,19,26,30,68].

### 6.4. Pharmacokinetics and In Vivo Evaluations

Preclinical studies are essential for assessing drug absorption, distribution, metabolism, and excretion (ADME), ensuring the therapeutic viability of CD–hydrogel formulations.

- Pharmacokinetic and Bioavailability Studies: Plasma concentration measurements, bioavailability assessments, and area under the curve (AUC) calculations in animal models (e.g., rats, rabbits) determine the efficiency of systemic drug absorption and clearance [3,20,33,48,54,61,64,76].
- Tissue Uptake and Distribution: To confirm localized drug retention and systemic exposure, tissue uptake studies are conducted in vaginal, ocular, and brain tissues, optimizing formulations for site-specific drug delivery [3,6,25,37,69,70].

### 6.5. Biocompatibility and Safety Evaluations

The safety and tolerability of CD–hydrogel formulations must be established before progressing to clinical studies.

- Cytotoxicity and Biocompatibility: In vitro biocompatibility is assessed through MTT assays, fibroblast viability studies, and histopathological analysis to ensure that formulations do not induce adverse cellular responses [3,19,20,31,35,44,71,77,79].
- Toxicity and Microbiological Assessments: Acute oral toxicity, oxidative stress markers, and microbiological evaluations are conducted to confirm that CD–hydrogel systems are non-toxic and suitable for human application [8,14,48,54,70,75,81].

### 6.6. Optimization and Formulation Development

CD–hydrogel formulations undergo systematic optimization using advanced experimental designs to enhance stability, drug loading, and therapeutic efficacy.

- Design of Experiments (DoE) and Formulation Optimization: The Box–Behnken design, Plackett-Burman screening, and factorial experimental designs are used to refine formulation parameters and optimize drug delivery performance [19,26,38,43,63,65].
- Drug Loading and Entrapment Efficiency: Maximizing drug loading and encapsulation efficiency ensures high drug retention within the hydrogel matrix, improving therapeutic efficacy and controlled release [17,19,29,36,44,48,76].
- Mechanical and Stability Testing: Hydrogel mechanical strength, spreadability, transparency, and polymer stability are evaluated under storage conditions to ensure the long-term integrity of formulations [16,24,43,53,57,76].

### 6.7. Biological Activity Studies

Beyond physicochemical and pharmacokinetic assessments, CD–hydrogel formulations must demonstrate therapeutic efficacy in disease-relevant models.

- Antimicrobial and Antifungal Activity: CD–hydrogel systems intended for infection management are evaluated against bacterial and fungal pathogens using microbiological assays and minimum inhibitory concentration (MIC) studies [3,13,14,22,53,70,73].
- Anti-Inflammatory and Anticancer Studies: The efficacy of CD–hydrogel formulations is assessed in inflammatory disease models (e.g., skin, ocular inflammation) and cancer cell lines (e.g., melanoma, breast cancer) to determine their therapeutic potential [1,4,21,41,71,77].
- Other Functional Assessments: Specialized applications, such as antioxidant capacity, sunscreen SPF evaluation, and enzymatic degradation studies, further validate CD–hydrogel formulations for dermal, transdermal, and targeted drug delivery [14,23,28,58,81].

## 7. Therapeutic Applications of CD–Hydrogel Hybrids

CD–hydrogel hybrids have been successfully employed in various therapeutic applications, including inflammation management, antifungal and antibacterial treatments, oncology, cardiovascular therapy, neurological disorders, and transdermal drug delivery. By incorporating CDs into hydrogels, formulations can be tailored to achieve targeted drug delivery, prolonged therapeutic effects, and reduced systemic toxicity.

### 7.1. Anti-Inflammatory and Pain-Relief Treatments

Lornoxicam, a potent analgesic and anti-inflammatory drug, has been incorporated into β-CD or HP-β-CD inclusion complexes in molar ratios ranging from 1:1 to 1:4. These complexes were formulated into a liquid crystalline gel containing Brij 97, glycerol (3:1), 10% Miglyol 812, and 40% water. The kneading method was used to prepare these inclusion complexes, optimizing drug solubility. The study identified that a 1:2 drug:CD molar ratio provided the best dissolution profile. Permeation studies on pig skin indicated low systemic absorption, enhancing localized pain relief while minimizing systemic side effects [1].

Another advanced formulation involves β-CD/epichlorohydrin polymer (EPI-β-CD) nanogels for an ibuprofen cutaneous delivery system. These nanogels, prepared without additional crosslinkers, exhibit a nanoscale structure ranging from 60 to 400 nm. The nanogel system significantly improved ibuprofen solubility and increased its permeation rate by up to 3.5 times compared to conventional reference gels. This enhancement suggests the formulation’s potential for improved topical and transdermal pain relief [5].

For transdermal iontophoresis, HP-β-CD inclusion complexes with piroxicam were developed into a specialized gel system. This formulation was evaluated using iontophoresis at 0.4 mA/cm^2^ for seven hours, leading to a 3.4-fold increase in drug flux compared to passive diffusion. Additionally, a significant amount of the drug remained in the skin, correlating well with the measured flux values. This approach demonstrates the feasibility of using iontophoresis to enhance localized drug delivery while controlling systemic absorption [15].

Another notable CD-based system includes dextran/β-CD macroporous cryogels synthesized via photochemical crosslinking in a frozen state. Curcumin, known for its anti-inflammatory and anticancer properties, was loaded into these cryogels by physical adsorption, achieving an encapsulation efficiency of 57.8%. The formulation provided sustained drug release over 72 h, without an initial burst effect. Furthermore, cytotoxicity studies revealed comparable efficacy to free curcumin, highlighting its potential for controlled dermal delivery and cutaneous T-cell lymphoma treatment [27].

For skin disorder treatment, β-CD-decorated thermoresponsive nanogels were synthesized using dendritic polyglycerol (dPG) as a crosslinker, to enhance dexamethasone delivery. This formulation achieved 2.5-times-higher epidermal drug delivery compared to commercial dexamethasone cream, and exhibited a remarkable 30-fold increase in dermal penetration. Furthermore, it demonstrated strong downregulation of thymic stromal lymphopoietin (TSLP), a key inflammatory cytokine, indicating superior therapeutic efficacy for inflammatory skin conditions [41].

In periodontal therapy, β-CD-based in situ forming gel (ISG) and microparticle (ISM) systems were designed for the sustained delivery of meloxicam, an anti-inflammatory drug used to manage periodontitis. These formulations were prepared using dimethyl sulfoxide (ISG system) and camellia oil with glyceryl monostearate (ISM system). The ISM system successfully extended drug release up to seven days, while minimizing burst release, demonstrating a controlled release mechanism governed by Fickian diffusion. These findings support the potential of β-CD-based ISG/ISM formulations in reducing inflammation and improving treatment adherence in periodontal disease [59].

### 7.2. Antifungal Therapies

For vaginal antifungal therapy, a β-CD-based mucoadhesive and thermosensitive gel was formulated using Pluronic F127 (20%), Carbopol 934 (0.2%), and hydroxypropylmethylcellulose (HPMC, 0.2%). Clotrimazole, a widely used antifungal agent, was incorporated into a 1:1 drug–CD inclusion complex. The gelation temperature was optimized, and drug release was evaluated in pH 5.5 citrate buffer. The β-CD complex significantly slowed drug release, ensuring prolonged therapeutic action. However, Carbopol 934 induced precipitation, which required further optimization. The final formulation maintained drug levels above the minimum inhibitory concentration (MIC), ensuring effective treatment against vaginal infections such as vaginitis [2].

Similarly, an HP-β-CD-based in situ vaginal gel was developed for voriconazole delivery. This formulation contained Poloxamer 407 (18%), Poloxamer 188 (5%), and HPMC (0.4%) to ensure mucoadhesive properties. Voriconazole was incorporated as a spray-dried HP-β-CD complex in a 1:2.5 molar ratio, achieving a gelation temperature of 31.7 ± 0.1 °C. The formulation exhibited controlled drug release, with 56.2 ± 0.44% released within 8 h, following a Fickian diffusion mechanism. Additionally, vaginal tissue uptake was significantly higher compared to non-CD gels, highlighting the potential of this system for enhanced localized antifungal therapy [6].

In ophthalmic applications, HP-β-CD and SBECD inclusion complexes were utilized to enhance fluconazole delivery in an ion-sensitive gel. This system incorporated gellan gum and kappa-carrageenan to improve bioadhesion and provide controlled drug release. The formulation exhibited low cytotoxicity in keratocyte cell assays, making it a promising candidate for treating fungal keratitis and other ocular fungal infections [7].

For transdermal antifungal therapy, a β-CD-fluconazole solid dispersion was integrated into an Aloe vera gel. Fluconazole-β-CD complexes were prepared via the solvent evaporation method, and an optimized gel formulation (GF1) was tested for release, permeability, and antifungal activity. Drug diffusion studies revealed a significantly higher release (96.92%) compared to a marketed fluconazole gel (89.24%). Furthermore, antifungal activity assessments demonstrated a 30 mm zone of inhibition, outperforming the commercial product (24 mm), confirming the formulation’s superior efficacy [53].

To enhance oral antifungal therapy, SH-β-CD inclusion complexes with fluconazole were formulated into mucoadhesive nanogels. These nanogels were synthesized via a Schiff base reaction using Carbopol 940 and gelatin, and their physicochemical properties were characterized through PXRD and FTIR analysis. The formulation demonstrated 18-fold improved mucoadhesion on buccal mucosa, with an 88% drug encapsulation efficiency. Drug release followed a first-order kinetic model, ensuring sustained antifungal activity against oral thrush [50].

For dermatological fungal infections, β-CD nanosponges crosslinked with N,N-carbonyldiimidazole were developed for the controlled delivery of econazole nitrate. The nanosponges were optimized using the Box–Behnken design, achieving an entrapment efficiency of 70.13%. These nanoparticles, with an average size of 421.37 ± 6.19 nm, were incorporated into a Carbopol 934 hydrogel. The final formulation exhibited superior skin permeability compared to the marketed product and remained stable under normal and accelerated conditions, supporting its use in the treatment of dermatophytosis and other cutaneous fungal infections [16].

Finally, for ophthalmic fungal infections such as fungal keratitis and endophthalmitis, fluconazole was complexed with HP-β-CD and incorporated into polymeric nanoparticles (Eudragit RS100, RL100; Evonik, Rohm GmbH Pharma Polymers (Darmstadt, Germany)) and niosomal vesicles. These nanoparticles exhibited an entrapment efficiency of 76.4%, a particle size of 151.1 nm, and a zeta potential of +40.1 mV. The system was further combined with an in situ thermosensitive gel for sustained ocular drug release. The formulation extended drug release over 24 h and significantly increased corneal permeability and bioavailability, as indicated by enhanced AUC_0–6_h values, making it an effective option for treating fungal eye infections [19].

### 7.3. Infections, Inflammation, and Glaucoma

A thermosensitive and pH-induced in situ ophthalmic gel incorporating HP-β-CD was developed for the delivery of ciprofloxacin hydrochloride, a broad-spectrum antibacterial agent used in treating ophthalmic infections. The drug was incorporated into a 1:1 HP-β-CD inclusion complex, confirmed via Fourier-transform infrared spectroscopy (FTIR) and differential scanning calorimetry (DSC). The formulation remained in liquid form at room temperature, but underwent gelation at ocular temperatures, ensuring prolonged retention in the ocular cavity. Drug release studies demonstrated sustained delivery over 8 h, effectively enhancing therapeutic efficacy while minimizing the need for frequent dosing [22].

For corticosteroid-based ocular inflammation treatment, γ-CD nanogels crosslinked through emulsification and solvent evaporation were developed, utilizing HPγ-CD as a stabilizing agent. Dexamethasone (25 mg/mL) was incorporated into these nanogels and tested in rabbit eyes. The results indicated prolonged drug retention, with a tear fluid concentration of 295 ± 59 µg/mL and an aqueous humor peak concentration of 136 ± 24 µg/mL—significantly higher than the reference Maxidex formulation (44.4 ± 7.8 µg/mL). This demonstrates the formulation’s ability to enhance corticosteroid delivery, ensuring longer-lasting anti-inflammatory effects with reduced systemic absorption [48].

A combined hydrogel system utilizing HP-β-CD and HPγ-CD was developed for dexamethasone acetate delivery in corneal inflammation treatment. These inclusion complexes were incorporated into three ophthalmic gel formulations—CELLUVISC, GEL-LARMES, and VISMED—and evaluated for osmolality and drug release. HP-β-CD-based gels released 90% of the drug within 2 h, while HPγ-CD-based gels released 60% in the same time frame. Compared to standard eye drops, these formulations demonstrated improved ocular retention, making them more effective for sustained anti-inflammatory therapy [32].

To address the challenges of protein deposition and burst release in ocular drug delivery, a novel polyvinyl alcohol (PVA) hydrogel system was developed using mono-methacrylated-β-CD (MA-β-CD) and methacrylated-PVA (PVAMA). This hydrogel was synthesized via UV polymerization, incorporating β-CD up to 30 wt%. The system was designed for the delivery of puerarin and acetazolamide, two therapeutic agents used in ocular treatment. Characterization studies confirmed an optimized swelling ratio, reduced burst release of acetazolamide, and decreased protein deposition. The hydrogel enabled sustained drug release for 15 days, significantly improving drug stability and ocular compatibility for long-term therapies such as glaucoma management [37].

### 7.4. Wound Healing and Regenerative Medicine

Allantoin, a well-known wound healing agent, was incorporated into β-CD and HP-β-CD inclusion complexes within a Carbopol-based gel. The 1:1 inclusion complex was optimized for gelation using 10% glycerol and triethanolamine, resulting in a mucoadhesive, self-healing gel with strong network formation and high hydrophilicity. The formulation exhibited prolonged biocompatibility and significantly enhanced antimicrobial activity against *Staphylococcus aureus* and *Candida albicans*, making it a promising candidate for wound care applications [13].

For antimicrobial wound dressings, a physically crosslinked chitosan (CS) and CM-β-CD hydrogel was developed to deliver berberine hydrochloride (BBH). The hydrogel was synthesized to improve the solubility and mechanical strength of BBH while ensuring sustained drug release. Antimicrobial tests demonstrated potent activity against *Escherichia coli*, *Staphylococcus aureus*, and *Candida albicans*, while biocompatibility studies confirmed the absence of cytotoxicity in NIH3T3 fibroblasts and HaCaT keratinocytes. This hydrogel system provides a safe and effective approach for treating infected wounds and promoting tissue regeneration [51].

For psoriasis treatment, a CD-based nanogel was designed to deliver babchi oil (*Psoralea corylifolia*) in a Carbopol gel. The nanostructured formulation contained babchi oil-loaded CD nanocarriers with an average particle size of 360.9 ± 19.55 nm. Viscosity and spreadability studies confirmed optimal gel characteristics for topical application. In in vivo studies, the formulation exhibited twice the antipsoriatic efficacy compared to a native babchi oil gel. Additionally, biochemical assays revealed enhanced superoxide dismutase (SOD) and glutathione (GSH) levels, along with reduced malondialdehyde (MDA) and nitric oxide (NO) levels, indicating strong antioxidant and anti-inflammatory effects. This novel nanogel system presents a promising approach for managing psoriasis and other inflammatory skin conditions [66].

### 7.5. Cancer Therapies

For HPV-induced cervical cancer, a thermosensitive mucoadhesive vaginal gel containing Pluronic F127, hyaluronic acid, Carbopol 934, and hydroxypropylmethylcellulose (HPMC) was formulated with a 5-fluorouracil (5-FU)-β-CD inclusion complex (1:1 molar ratio). The formulation was optimized for gelation temperature and drug release profile, achieving complete drug release. β-CD accelerated the release rate, except in the presence of Carbopol 934, which modulated the drug release kinetics. Cytotoxicity studies confirmed that the formulation exhibited comparable anticancer activity to free 5-FU, indicating its potential for localized cervical cancer therapy [35].

For colorectal cancer, a thermoreversible gelling film was developed using poloxamer 407, poloxamer 188, and HP-β-CD to deliver 5-FU. The inclusion complex (1:1 molar ratio) significantly improved drug solubility and transport efficiency across rectal membranes. Compared to conventional formulations, the system enhanced drug transport efficiency by 7.3-fold and cellular uptake by 5.4-fold, while demonstrating prolonged drug release and no signs of rectal irritation, making it a promising approach for colorectal cancer treatment [25].

In the field of nanomedicine, paclitaxel-loaded nanoparticles were developed using TPGS(2k), gelatin-grafted CD, and hyaluronic acid-grafted CD to enhance oral bioavailability and tumor targeting. The nanoparticles were prepared via ultrasonic crushing, achieving an average size of 253.57 ± 2.64 nm and an encapsulation efficiency of 61.77 ± 0.47%. The formulation exhibited sustained drug release and improved paclitaxel bioavailability by 227.21%. The presence of hyaluronic acid facilitated targeted uptake via CD44 receptor-mediated endocytosis, significantly enhancing cellular uptake and therapeutic efficacy against tumors [36].

For intratumoral therapy, Pluronic F127 and P123 hydrogels incorporating RM-β-CD and ethanol were optimized for beta-lapachone delivery. This temperature-sensitive gel depot provided a 50-fold increase in drug solubility and exhibited a diffusion coefficient ranging from 9 to 69 µg·cm^−2^. While ethanol enhanced drug release, it also weakened the gel structure, highlighting the need for formulation balance. This delivery system presents a promising method for direct intratumoral chemotherapy with controlled drug release [24].

A novel injectable nanocomposite paste was designed using gelatin (Gel) and β-CD-grafted chitosan nanoparticles (CS-g-CD NPs) for doxorubicin delivery in postoperative tumor therapy. This shear-thinning and self-healing paste exhibited pH-responsive drug release in the tumor microenvironment, ensuring sustained drug action. In vivo studies demonstrated enhanced tumor cell inhibition and effective prevention of tumor recurrence in mice, validating its potential as a localized, minimally invasive cancer therapy [21].

For breast cancer treatment, a CD nanosponge hydrogel system was developed by crosslinking a CD-nanosponge system with pyromellitic dianhydride and incorporating curcumin and resveratrol into a carbomer-propylene glycol hydrogel. This formulation was optimized using the Box–Behnken design, achieving a 5-fold increase in curcumin photostability and a 7-fold increase for resveratrol. Drug release studies demonstrated a 10-fold increase in curcumin release and a 2.5-fold increase in resveratrol release. The combination of these compounds exhibited strong synergistic cytotoxicity against MCF-7 breast cancer cells (combination index, CI = 0.29) and significantly enhanced transdermal permeation, offering a non-invasive alternative for breast cancer therapy [65].

### 7.6. Neurological and Psychiatric Treatments

For intranasal anticonvulsant therapy, a mucoadhesive, thermosensitive in situ gel was developed using poloxamer, chitosan glutamate, sodium hyaluronate, and RAMEB to deliver clonazepam. The formulation exhibited a gelation temperature of 29–30.5 °C and a mucoadhesion time of 6 h, ensuring prolonged nasal residence. Optimized via Design of Experiments (DoE), the gel enhanced drug release and permeation while maintaining low cytotoxicity, making it a promising alternative for epilepsy management, with improved patient compliance [26].

For schizophrenia treatment, a thermoresponsive in situ nasal gel containing poloxamer 407, polyvinylpyrrolidone K30 (PVP K30), and HP-β-CD was formulated for the delivery of asenapine maleate. The HP-β-CD inclusion complex (1:1 molar ratio) was incorporated into the nasal gel, achieving gelation at 29–34 °C with 23% *w*/*v* poloxamer 407. This system demonstrated rapid drug diffusion, with 99.1% of the drug released within 120 min. Pharmacokinetic studies revealed a 2.5-fold increase in bioavailability compared to the oral formulation, with a peak plasma concentration (*C_max_*) of 9 ng/mL (nasal gel) versus 5.5 ng/mL (HP-β-CD solution) and 2 ng/mL (oral administration). These findings suggest the nasal gel as an effective non-invasive alternative for schizophrenia therapy, with improved drug absorption and faster onset of action [28].

For post-traumatic stress disorder (PTSD) management, an armodafinil-loaded methylcellulose (MC) hydrogel was developed using an armodafinil-(HP-β-CD) inclusion complex. The complex was prepared via lyophilization at a 1:1 molar ratio, achieving an encapsulation efficiency of 90.98% and a 21-fold increase in solubility. The hydrogel formulation was optimized for controlled drug release, leading to improved brain targeting and extended therapeutic effects. In vivo studies demonstrated that an 11-day treatment regimen significantly improved PTSD symptoms in mice, highlighting the formulation’s potential for long-term cognitive enhancement and mental health treatment [31].

For sustained schizophrenia management, a HP-β-CD-paliperidone inclusion complex was incorporated into a pH-responsive in situ nasal gel containing carbopol 934 and hydroxypropyl methylcellulose (HPMC). This formulation was evaluated for nasal mucosal permeation and histopathological safety. The results indicated enhanced drug permeation across the nasal mucosa, sustained drug release, and the absence of mucosal toxicity, making it a viable alternative for long-term schizophrenia treatment with improved drug absorption and reduced systemic side effects [74].

### 7.7. Diabetes and Metabolic Disorders

For oral insulin delivery, M-β-CD-complexed insulin was encapsulated into polymethacrylic acid (PMAA) hydrogel microparticles. The insulin-MCD complex was incorporated into a poly(methacrylic acid)-chitosan-PEG (PCP) matrix, which was extensively evaluated for drug loading, permeability, and in vivo efficacy. Permeability studies using Caco-2 cell monolayers demonstrated enhanced intestinal absorption of insulin. In vivo studies in diabetic rats confirmed significant glucose reduction, highlighting the improved bioavailability of oral insulin and its potential to replace invasive insulin administration methods [30].

A further advancement in oral insulin therapy involved the development of CM-β-CD-grafted carboxymethyl chitosan (CMC) hydrogel microparticles. These hydrogels were synthesized via water-soluble carbodiimide crosslinking, allowing for pH-triggered release of insulin in the intestinal environment. The formulation demonstrated sustained glucose reduction over a period of 6 to 12 h, ensuring prolonged therapeutic efficacy. Additionally, insulin remained stable post-release, maintaining its bioactivity throughout the controlled delivery process. This system offers a promising approach for optimizing glucose regulation while minimizing frequent insulin dosing [67].

### 7.8. Inflammatory Bowel Disease, Cancer, and Biologic Therapies

Colon drug delivery (CDD) directs drugs to the colon for local treatment or controlled systemic absorption. Corticosteroids, commonly used for conditions like inflammatory bowel disease (IBD), often cause systemic side effects. To address this, an inulin- β-CD (INUCD) bioconjugate was developed, combining inulin for colon-targeted degradation and β-CD for drug solubilization [82]. Characterization studies and in vitro tests confirmed its ability to deliver drugs locally while reducing systemic absorption, making it ideal for prolonged therapies. Many biologics suffer from poor solubility and bioavailability, limiting their therapeutic effectiveness. CD inclusion complexes significantly improve drug solubility, as demonstrated with viridicatin derivatives, which showed a fourfold increase in selectivity for diseased cells [83]. Similarly, beta-lapachone (β-lap), an anticancer agent, achieved a 16-fold solubility increase when complexed with HP-β-CD, improving its cytotoxic efficiency and safety profile in in vivo studies [84]. Beyond colon-specific delivery, cyclodextrins facilitate inhalable biologic therapies. A spray-dried formulation of monoclonal antibodies with HP-β-CD and L-leucine demonstrated >80% aerosol emission and preserved antigen-binding potency, offering a non-invasive, self-administered alternative for asthma treatment [85].

Many cyclodextrin hydrogel systems have been developed primarily for small-molecule drug delivery, including applications in ophthalmic drug delivery [29], oral insulin delivery [30,67], berberine hydrochloride release [51], and acyclovir solubility enhancement [80]. While these hydrogels excel in improving drug solubility, controlled release, and bioavailability, their application in biologics, such as proteins, peptides, and nucleic acids, remains less explored within the cited studies. Some advancements have been made in systemic drug delivery, such as paclitaxel-loaded cyclodextrin nanoparticles for oral tumor-targeted therapy [36], but further refinement is needed for broader precision-targeted applications, including cancer and diabetes treatment. Additionally, rectal thermoreversible gelling films incorporating cyclodextrin inclusion complexes have been developed for localized colorectal cancer treatment, demonstrating prolonged drug release and enhanced cellular uptake [25]. Overall, cyclodextrin-based hydrogels offer potential for next-generation therapeutic systems by improving drug solubility, controlled release, and bioavailability, though more research is needed to extend their use in biologic drug delivery.

Table 5 categorizes drugs and therapeutic agents based on their CD formulations, highlighting key applications and reference numbers. Non-Steroidal Anti-Inflammatory Drugs (NSAIDs) like ibuprofen and piroxicam are commonly formulated with β-CD, γ-CD, and HP-β-CD for enhanced solubility in topical and transdermal gels. Antifungal agents such as fluconazole use SBECD and HP-β-CD for systemic and localized fungal infections. Ophthalmic drugs leverage CDs for sustained ocular release, while anticancer drugs like paclitaxel benefit from CD nanosponges for targeted chemotherapy. Antibiotics and antiviral drugs utilize mucoadhesive and pH-sensitive hydrogels for controlled release. Antipsychotic drugs leverage CDs for nasal and transdermal delivery, improving bioavailability. Cardiovascular drugs such as statins incorporate β-CD-based nanogels for cholesterol management. Hormone therapies utilize M-β-CD and HP-γ-CD for improved delivery. Lastly, natural therapeutics like curcumin employ CD nanosponges to enhance bioavailability and therapeutic efficacy.

## 8. Advancements in CD–Hydrogel Drug Delivery

### 8.1. Enhanced Drug Solubility and Bioavailability

One of the most significant advantages of CD–hydrogel systems is their ability to improve the solubility of poorly water-soluble drugs, increasing their bioavailability and therapeutic potential. CD inclusion complexes, particularly HP-β-CD, β-CD, and γ-CD, have demonstrated superior drug solubility across a wide range of pharmaceutical formulations. Studies have shown marked improvements in the solubility of paliperidone, sildenafil, insulin, lornoxicam, rosuvastatin, ciprofloxacin, ketoconazole, azithromycin, asenapine, dexamethasone, fluconazole, berberine hydrochloride, peppermint, calcipotriol, rutin, 5-fluorouracil, paclitaxel, minoxidil, curcumin, dexibuprofen, valacyclovir, artemether, and acyclovir, leading to better therapeutic efficacy and patient outcomes [1,3,5,17,18,19,22,23,25,28,32,34,35,36,42,43,44,47,51,52,53,54,63,65,67,69,72,74,79,80,81].

In addition, nanogels, mucoadhesive hydrogels, in situ gelling formulations, hydrogel-forming microneedle patches and polypseudorotaxane hydrogels have significantly improved drug retention and permeability, particularly in ocular, transdermal, and oral drug delivery applications [3,4,5,19,20,22,31,45,64,76]. These formulations enhance drug bioavailability by increasing residence time at the target site, while minimizing systemic side effects.

Another critical advancement is the stabilization of photolabile compounds. Studies on curcumin, resveratrol, babchi oil (Psoralea corylifolia), and fluconazole have demonstrated that CD-based nanogels and nanosponge formulations prevent drug crystallization and degradation, thereby extending shelf life and improving treatment efficacy for transdermal, topical, and cancer therapies [21,24,44,50,65,66].

Finally, CD–hydrogel formulations have substantially enhanced systemic bioavailability. For example, hydrogel-forming microneedle patches and nanogel formulations have improved bioavailability by more than 100% for sildenafil citrate, 227.21% for paclitaxel, 20-fold for telmisartan and 18-fold for fexofenadine, presenting novel opportunities for non-invasive systemic drug delivery [36,42,62,76].

### 8.2. Controlled and Sustained Drug Release

A key advantage of CD–hydrogel formulations is their ability to provide controlled, sustained, and site-specific drug release, leading to prolonged therapeutic effects and improved patient compliance. Mucoadhesive and thermosensitive hydrogels, in situ gels, and microneedle systems have been extensively studied for their ability to enhance localized drug retention and reduce dosing frequency [2,4,6,7,22,25,33,35,38,43,50,54,55,59,64,68].

**Figure 2:**
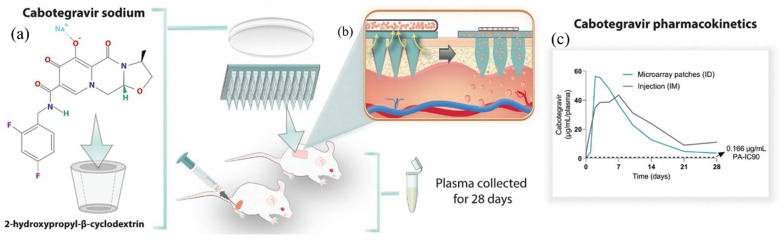
A graphical representation showing the following: (**a**) CAB-Na was combined with HP-β-CD to formulate a suitable tablet reservoir that dissolved rapidly and completely when situated atop a swollen HF-MAP. (**b**,**c**) Intradermal delivery of this anti-HIV therapeutic was achieved over 24 h in rats in vivo, and drug pharmacokinetics were studied over the following 28 days, revealing that the formulated HF-MAP device had a long-acting delivery profile. Adopted with permission [61].

Hydrogel-forming microneedle patches have successfully enabled sustained-release drug delivery, including long-acting HIV prophylaxis (cabotegravir sodium), transdermal antihypertensive therapy (telmisartan), erectile dysfunction therapy (sildenafil citrate) and schizophrenia treatment (risperidone) [42,45,61,76]. Hydrogel-forming microarray patches (HF-MAPs) are designed to be self-administered and self-disabling, preventing the generation of contaminated sharps waste. The use of HP-β-CD to enhance the solubility of cabotegravir sodium (CAB-Na), and its impact on intradermal delivery via HF-MAPs, is also illustrated in Figure 2 [61]. These systems offer minimally invasive drug administration, reducing patient discomfort and improving adherence to treatment regimens.

Periodontal and ophthalmic applications have also benefited from CD-based hydrogel technology, with formulations demonstrating extended antibiotic and anti-inflammatory drug release. Notably, meloxicam hydrogels for periodontitis provided sustained drug release for 7 days, while ocular antibiotic CD–hydrogel formulations released drugs continuously for two weeks, enhancing long-term therapeutic effectiveness [39,59].

### 8.3. Enhanced Drug Retention, Permeation, and Therapeutic Efficacy

Ophthalmic CD–hydrogel formulations, for example, have improved corneal penetration, intraocular retention, and antifungal efficacy, especially in treatments involving ciprofloxacin, dexamethasone, flurbiprofen, and josamycin [3,4,19,22,29,32,39,48,49,57,64,69,70,72].

In transdermal delivery, CD-functionalized nanogels, microneedles, and supramolecular hydrogels have shown significant success in enhancing dermal drug penetration and bioavailability. Curcumin, resveratrol, dexamethasone, telmisartan, and piroxicam formulations have exhibited enhanced skin permeability and systemic absorption, making them suitable for non-invasive therapies for inflammation, pain, and cancer [15,17,40,41,44,65,76,77,78].

CD inclusion complexes have also facilitated brain-targeted drug delivery via intranasal administration, increasing drug permeability across the blood–brain barrier. This approach has significantly improved bioavailability in formulations for PTSD, schizophrenia, cerebral malaria, and neurodegenerative disorders [26,31,63,74].

Finally, CD-nanogels and hydrogel microparticles have increased oral insulin transport and bioavailability, overcoming one of the most significant challenges in diabetes treatment—intestinal absorption barriers [30,58,67].

### 8.4. Biocompatibility and Reduced Toxicity

A major advantage of CD–hydrogel formulations is their high biocompatibility and low toxicity, making them suitable for long-term applications in ophthalmic, oral, and transdermal drug delivery. Studies confirm that CD–hydrogel formulations show no toxicity or irritation in in vivo models, ensuring safe and effective pharmaceutical applications [5,8,13,18,20,25,39,42,46,47,48,50,51,54,57,63,67,79].

Hydrogel-based microneedles and transdermal patches have shown superior safety profiles, eliminating irritation-causing solvents used in conventional formulations for alopecia, erectile dysfunction, and cardiovascular treatments [42,47,68].

**Figure 3:**
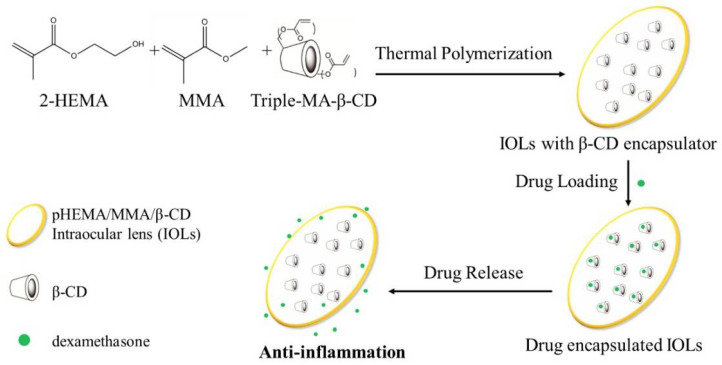
An illustration of pHEMA/MMA/β-CD intraocular lens (IOL) with the capability of maintaining the sustained release of anti-inflammatory drugs like dexamethasone. Adopted with permission [57].

CD-functionalized intraocular hydrogel lenses and contact lenses have demonstrated long-term safety and strong antimicrobial activity, particularly against Pseudomonas aeruginosa and Staphylococcus aureus, making them promising options for post-cataract surgery drug delivery [39,57]. In ocular systems, β-CD hydrogels containing poly(2-hydroxyethyl methacrylate-co-methyl methacrylate) (p(HEMA-co-MMA)) were designed and prepared as intraocular lens (IOL) biomaterials, which demonstrated enhanced swelling, facilitating the sustained release of dexamethasone, as shown in Figure 3. A series of pHEMA/MMA/β-CD copolymers with varying β-CD ratios were synthesized using thermal polymerization. The resulting polymers exhibited high transmittance at visible wavelengths and demonstrated good biocompatibility with mouse connective tissue fibroblasts. Drug loading and release studies showed that incorporating β-CD into the hydrogels enhanced loading efficiency and facilitated sustained drug release [57].

Additionally, CD-emulgel formulations have significantly reduced drug-associated side effects, as seen in calcipotriol for psoriasis and estradiol for hormone therapy, improving therapeutic efficacy and patient adherence [33,52].

Table 6 explores advanced applications and innovations in CD-based hydrogels, highlighting their impact on drug delivery, regenerative medicine, and scalable production. Synergistic CD use (e.g., HP-β-CD with γ-CD) improves solubility, bioavailability, and ocular retention, while γ-CD metal–organic frameworks (γ-CD-MOFs) enhance photostability and targeted hydrophobic drug delivery. Hybrid polymers combining natural (chitosan) and synthetic (PEG, Pluronic F127) components improve gelation, strength, and sustained release. Stimuli-responsive hydrogels enable spatiotemporal drug release, particularly for oncology and mucosal therapies. Nanosponges and nanogels provide enhanced stability and sustained drug release, while bioactive CD hydrogels exhibit antimicrobial and antioxidant properties for wound healing and tissue repair. Non-invasive technologies, such as microneedle-integrated hydrogels, improve patient compliance in chronic disease treatments. Scalable and sustainable production via spray-drying and freeze-drying ensures reproducibility, with eco-friendly approaches aligning with regulatory standards. Regenerative medicine benefits from the use of drug-eluting CD hydrogels in tissue scaffolds and wound healing. Comparative performance studies show that CD hydrogels outperform commercial formulations, particularly in antifungal and transdermal applications. Emerging frontiers include gene therapy, neurodegenerative diseases, and microneedle-based melanoma treatment. Optimized cancer drug delivery through pH-sensitive CD hydrogels and combination therapies enhances localized targeting while minimizing toxicity.

## 9. Challenges and Limitations of CD–Hydrogel Systems

### 9.1. Complex Formulation and Manufacturing Challenges

One of the primary obstacles in the development of CD–hydrogel systems is the complexity of formulation design and optimization. Achieving the optimal drug–CD ratio for effective complexation requires extensive optimization, as different drugs exhibit unique binding affinities to CD derivatives, impacting solubility, stability, and release kinetics [1,2,5,34,40,45,46,76]. Additionally, the use of crosslinking agents in nanogels and hydrogels can significantly alter drug release profiles, sometimes leading to reduced sustained release potential [5].

The fabrication of hydrogel-forming microneedle patches, nanosponges, polypseudorotaxane hydrogels, and cryogels presents scalability issues, as these systems require precise synthesis techniques and advanced polymer engineering [17,27,50,51,61,64,65,77]. The large-scale production of mucoadhesive hydrogels and nanogels is also limited by batch-to-batch variability and inconsistent adhesion properties, impacting industrial feasibility and reproducibility [28,30,31,35,36,50,67].

Furthermore, thermosensitive and supramolecular hydrogels require precise temperature control during formulation and storage, posing stability and transport challenges [26,28,55,62,63,69,78].

### 9.2. Stability and Variability in Drug Release Profiles

Another major challenge in CD–hydrogel formulations is variability in drug release profiles, often resulting in burst release, inconsistent sustained delivery, or incomplete drug diffusion. pH-sensitive, iontophoretic, and mucoadhesive drug delivery systems exhibit unpredictable release rates, necessitating further optimization to ensure uniform and reproducible therapeutic effects [7,14,15,16,21,22,46,54,59,74].

Ophthalmic and nasal CD–hydrogel systems face challenges in achieving prolonged retention and uniform release. For example, some dexamethasone-CD complexes exhibited rapid release with HP-β-CD, while HP-γ-CD showed incomplete drug diffusion, highlighting the need for fine-tuning [32]. Similarly, estradiol in situ gelling nasal inserts, formed by complexation with M-β-CD, exhibited lower peak serum levels, which, while reducing side effects, necessitated dose adjustments to maintain therapeutic efficacy [33].

In transdermal and implantable systems, hydrogel-based antibiotic delivery demonstrated inconsistent release patterns, where ciprofloxacin exhibited modulated diffusion, but vancomycin showed no interaction with CD, indicating drug-specific limitations [46]. Mucoadhesive hydrogels also displayed inconsistencies, as excessive adhesion strength slowed drug diffusion, reducing bioavailability and delaying therapeutic effects [2,6,13,50].

### 9.3. Limited In Vivo Validation and Clinical Translation

Despite extensive preclinical studies, most CD–hydrogel systems lack large-scale clinical validation, with the majority of data derived from in vitro and small-animal models. This gap significantly limits the real-world applicability of these formulations, delaying regulatory approval and commercialization [8,14,15,17,18,19,21,30,31,35,36,42,50,52,56,57,58,71,73,77].

Long-term toxicity studies remain inadequate, particularly for novel CD-based hydrogel systems, such as metal–organic frameworks (MOFs) and polypseudorotaxane hydrogels, for which potential immunogenicity, accumulation, and chronic exposure risks have not been fully evaluated [17,64]. Additionally, oral insulin delivery via CD-based microparticles remains challenging, as batch-to-batch variability and gastrointestinal degradation issues affect reproducibility and efficacy [30,67].

### 9.4. Regulatory Barriers and Commercialization Hurdles

The complexity of CD–hydrogel formulations, coupled with the need for long-term safety data, presents significant regulatory hurdles. The lack of standardized regulatory pathways for CD-based nanocarriers and hydrogel drug delivery systems makes it difficult to gain FDA or EMA approval, slowing down market entry and clinical adoption [25,30,31,36,51].

Oral peptide and protein drug delivery systems—such as oral insulin and protein-loaded nanoparticles—face particularly high regulatory scrutiny, given the challenges associated with intestinal absorption, enzymatic degradation, and long-term metabolic effects [30,58,67]. Additionally, novel transdermal and bone implant CD-based hydrogel therapeutics remain under evaluation, requiring extensive clinical trials to confirm efficacy and safety before approval [46].

Scalability remains a concern, particularly for microneedle-based transdermal and intranasal hydrogel systems, which require advanced manufacturing processes to ensure batch consistency, sterility, and large-scale production feasibility [31,42,45,76].

## 10. Future Directions in CD–Hydrogel Research and Development

### 10.1. Development of Next-Generation Smart Hydrogels

The next generation of CD–hydrogels should focus on stimuli-responsive and multifunctional materials to enable personalized, site-specific, and on-demand drug delivery. Advances in pH-sensitive, enzyme-activated, redox-sensitive, and thermoresponsive hydrogels will allow for precise control of drug release based on physiological conditions [3,4,6,14,15,16,18,21,22,26,31,44,54,55,59,67,70,72]. Additionally, hybrid biomaterial–CD hydrogel systems should be explored for applications in bone regeneration, wound healing, and controlled antibiotic release [46,56,57]. These systems could incorporate bioactive peptides, antimicrobial agents, or growth factors, making them ideal for regenerative medicine and chronic disease management [13,20,41,51]. To further optimize stability and sustained drug release, self-healing and shear-thinning hydrogels should be developed, particularly for ocular therapeutics, localized chemotherapy, postoperative wound healing, and tissue regeneration [21,24,37].

### 10.2. Enhancing Drug Loading and Optimizing Release Kinetics

A major focus of future research should be improving drug loading efficiency and optimizing release kinetics to balance immediate and sustained drug release. Hybrid CD-nanogel systems that combine different CD derivatives could enhance drug solubility while maintaining long-term controlled release [5,36,38]. Layered drug delivery systems, integrating nanogels and mucoadhesive hydrogels, could enable dual-release profiles, making them ideal for chronic disease treatments [4,43]. These systems should also be tailored for hormonal therapies, probiotics, and immune-modulating drugs, enhancing oral and vaginal drug delivery applications [35,50]. For cancer therapy, cryogel-based hydrogels should be explored for localized chemotherapy in solid tumors, potentially reducing systemic toxicity and improving patient outcomes [27].

### 10.3. Expanding Applications in Neurology, Oncology, and Transdermal Therapy

CD–hydrogel drug delivery systems should be further optimized for neurodegenerative diseases, cancer therapy, and chronic conditions. In neurological disorders, intranasal CD–hydrogels could be explored for the treatment of post-traumatic stress disorder, cerebral malaria, and schizophrenia, leveraging their ability to bypass the blood–brain barrier and enhance drug delivery to the central nervous system [26,31,63,74]. For oncology applications, paclitaxel-loaded CD nanoparticles should be investigated for treating pancreatic and metastatic cancers, utilizing tumor-specific targeting ligands to improve therapeutic efficacy and reduce toxicity [36]. Transdermal CD–hydrogel cancer therapies should also be optimized for localized melanoma treatment, offering a non-invasive alternative to systemic chemotherapy [44]. Additionally, CD-based hydrogels could be expanded into dermatological applications, such as psoriasis, eczema, rosacea, and acne treatments, by leveraging their penetration-enhancing and anti-inflammatory properties [41,66].

### 10.4. Bridging the Gap Between Preclinical Research and Clinical Trials

To facilitate the widespread adoption of CD–hydrogel drug delivery systems, in-depth pharmacokinetics and biodistribution studies are necessary to translate ex vivo and animal findings into clinical applications [8,14,17,77]. A major limitation in CD–hydrogel development is the lack of large-scale clinical studies. Rigorous, well-controlled human trials are required to confirm the safety, efficacy, and long-term stability of CD–hydrogel formulations, particularly in ophthalmic, transdermal, oral, and nasal drug delivery systems [19,25,28,33,34,36,42,44,45,49,50,54,56,59,66,71]. To streamline regulatory approvals, regulatory pathways should be refined to accelerate the commercialization of advanced CD-based drug delivery systems while maintaining safety and efficacy standards [7,13,18,32,35,38,46,51,64,75,79].

### 10.5. Optimization of Oral Peptide and Hormone Delivery Systems

Significant efforts should be directed toward optimizing oral peptide and hormone delivery, particularly for insulin, testosterone, progesterone, and growth hormones. CD-based oral insulin formulations should be improved for reproducibility and enhanced intestinal permeability, potentially integrating microemulsion or nanogel technologies to ensure efficient absorption [30,67]. Similarly, vaginal and nasal hormone therapies should be expanded to include testosterone, progesterone, and peptide-based treatments, leveraging mucoadhesive and thermosensitive hydrogel platforms for sustained hormone release [33,38]. Biotin-grafted CD nanoparticles should also be optimized for oral protein drug delivery, expanding applications to monoclonal antibodies, vaccines, and other large-molecule therapies [58].

### 10.6. Enhancing Stability and Commercial Scalability

For CD–hydrogels to be commercially viable, their long-term storage stability and large-scale production processes must be optimized. Nano-organogels and CD-functionalized nanogels should be modified to ensure long-term stability without aggregation or burst release, making them feasible for industrial production [40,60,81]. To ensure predictable and sustained drug release, CD–hydrogel formulations should be modified to prevent burst effects, particularly in ophthalmic, buccal, and transdermal drug delivery applications [3,22,60,71,73,77]. Additionally, microneedle-based transdermal delivery systems should be further explored for peptide and protein drugs, offering pain-free and efficient systemic drug administration [45,76].

## 11. Conclusions

CD–hydrogel hybrids represent a transformative step in drug delivery, integrating solubility enhancement, controlled drug release, and biocompatibility into a single system. These hybrids have demonstrated promising applications across ophthalmic, transdermal, oral, and injectable formulations, but challenges such as batch-to-batch variability, stability concerns, and regulatory complexities must be addressed. Advanced stimuli-responsive hydrogels and functionalized CD-based materials offer new possibilities for targeted and personalized drug therapy. To maximize their impact, multidisciplinary research efforts must focus on optimizing formulation scalability, conducting rigorous clinical studies, and refining regulatory pathways. With continued innovation, CD–hydrogel hybrids are poised to become a cornerstone of next-generation pharmaceutical drug delivery systems, unlocking safer, more effective, and patient-friendly treatments.

## Figures and Tables

**Table 1:** Cyclodextrin types and pharmaceutical applications.

Type of Cyclodextrin	Key Features and Applications	Key Formulation Characteristics	References
Beta-cyclodextrin (β-CD)	Most widely used; enhances drug solubility, stability, and controlled release; applied in wound healing, transdermal, and oral gels.	Forms strong inclusion complexes with hydrophobic drugs, improving solubility and stability.	[1,5,13,14,20,24,44,47,53,54,55,56,57,58,59,60]
Hydroxypropyl-beta-cyclodextrin (HP-β-CD)	Improves drug permeation, bioavailability, and mucoadhesion; widely used in thermosensitive, vaginal, ophthalmic, and nasal gels.	Provides higher aqueous solubility than β-CD, making it ideal for mucoadhesive and injectable hydrogels.	[3,4,15,19,22,23,25,28,31,38,43,45,61,62,63]
Gamma-cyclodextrin (γ-CD)	Effective in ophthalmic, nanogel, and sustained drug release formulations; improves solubility and stability for poorly soluble drugs.	Has a larger cavity size, allowing encapsulation of larger hydrophobic molecules.	[17,34,48,49,64]
Cyclodextrin nanosponge (CDNS)	Advanced crosslinked structures provide extended drug release, high encapsulation efficiency, and improved drug stability.	Forms nanostructured polymeric networks, allowing higher drug loading capacity.	[16,65,66]
Sulfobutyl ether-beta-cyclodextrin (SBECD)	Enhances solubility and stability in antifungal, ophthalmic, and systemic drug delivery applications.	Provides high aqueous solubility and is often used in parenteral formulations.	[7]
Carboxymethyl-beta-cyclodextrin (CMCD)	Used in pH-responsive oral and transdermal hydrogels; supports controlled drug release for antibacterial and protein therapies.	Forms pH-sensitive hydrogels, enabling controlled and site-specific drug release.	[51,67]
Methyl-beta-cyclodextrin (Mβ-CD)	Applied in nasal, topical, and transdermal drug formulations to enhance drug absorption.	Improves membrane permeability, making it effective for nasal and dermal applications.	[33,68]
Hydroxypropyl-gamma-cyclodextrin (HPγ-CD)	Primarily used in ophthalmic and vaginal hydrogels; increases solubility and drug bioavailability.	Similar to HP-β-CD, but with a larger cavity size, accommodating bigger molecules.	[32,48]
Alpha-cyclodextrin (α-CD)	Applied in supramolecular poloxamer-based hydrogels for ocular drug delivery and controlled release.	Has the smallest cavity size, making it suitable for small-molecule drug encapsulation.	[69,70]
Modified cyclodextrins (Captisol, RAMEB, etc.)	Specialized derivatives tailored for targeted drug release, solubility enhancement, and bioavailability improvement.	Designed for high-affinity drug binding and customized inclusion complexes.	[26,52,71]

**Table 2:** Key hydrogel polymers in drug delivery.

Type of Hydrogel Polymer	Key Features and Applications	Key Formulation Characteristics	References
Poloxamer 407/Pluronic F127	Most commonly used for thermosensitive hydrogels; provides mucoadhesion, in situ gelation, and enhanced drug release.	Gel forms liquid at room temperature and solidifies at body temperature.	[2,6,19,24,26,28,35,43,44,55,62,70,71,72]
Carbopol	Widely applied in mucoadhesive formulations; enables controlled release in ophthalmic, transdermal, and vaginal gels.	Forms highly viscous gels that enhance bioadhesion.	[2,4,13,16,35,43,50,55,66,68,74]
Hydroxypropyl methylcellulose (HPMC)	Enhances viscosity, gel strength, and bioadhesion; commonly used in ophthalmic, vaginal, and buccal applications.	Provides sustained drug release; often combined with thermogelling agents.	[6,19,23,25,33,35,63,69,72,74,75]
Chitosan	Improves mucoadhesion, controlled drug release, and bioavailability; frequently used in nasal, ocular, and oral delivery.	Forms pH-sensitive gels that dissolve at acidic pH.	[19,20,21,26,29,30,51,54,58,62,67]
Polyvinyl alcohol (PVA)	Provides biocompatibility, mechanical strength, and controlled release properties; used in ophthalmic and transdermal hydrogels.	Forms elastic, mechanically strong films for prolonged drug release.	[37,45,76]
Gelatin	Used for biodegradable and injectable formulations; applied in microneedles and self-healing hydrogels.	Crosslinked gelatin forms hydrogels that degrade in vivo.	[21,36,50,77]
CD-based nanosponges	Enhances drug stability and sustained release; often incorporated in topical and transdermal gels.	Forms crosslinked networks for extended drug entrapment.	[16,17,65]
Polyethylene glycol (PEG)	Applied in crosslinked hydrogels for extended drug release and controlled solubility.	Often chemically crosslinked to enhance stability and swelling.	[30,71,78]
Hydroxyethyl methacrylate (HEMA)	Used in contact lens drug delivery and ophthalmic formulations.	Provides high water content and oxygen permeability for ocular applications.	[29,39,57]
Methylcellulose (MC)	Provides temperature-sensitive properties; applied in ophthalmic and transdermal hydrogels.	Forms reversible thermogels that transition at physiological temperatures.	[31]

**Table 3:** Techniques for CD–hydrogel preparation.

Process or Technique	Key Features and Applications	Common Cyclodextrins Used	Key Technical Data	References
Kneading	Frequently used for preparing drug–CD inclusion complexes; enhances solubility and bioavailability.	Beta-cyclodextrin, hydroxypropyl-beta-cyclodextrin	Drug–CD molar ratio of 1:1 to 1:4; kneading with minimal solvent (ethanol, water, or acetone); results in solid inclusion complex	[1,13,75]
Solvent evaporation	Produces stable inclusion complexes by dissolving CD and drug in a common solvent, followed by solvent removal.	Beta-cyclodextrin, sulfobutyl ether-beta-cyclodextrin	Solvent selection: ethanol, methanol, or acetone; evaporation temperature: 40–60 °C; complexation verified by FTIR, DSC, and PXRD	[48,53,75]
Freeze-drying (lyophilization)	Used for enhancing drug stability and solubility; commonly applied in ophthalmic and injectable hydrogels.	Hydroxypropyl-beta-cyclodextrin, methyl-beta-cyclodextrin	Lyophilization cycle: −50 °C to 25 °C; drug–CD complex dissolved in aqueous medium and frozen before sublimation	[33,68]
Spray-drying	Produces fine powders of drug–CD complexes, increasing dissolution and bioavailability.	Hydroxypropyl-beta-cyclodextrin, hydroxypropyl-gamma-cyclodextrin	Inlet temperature: 80–120 °C; solvent system: water–alcohol; particle size: 200–500 nm	[6]
Nanogel formation (free radical polymerization)	Used for crosslinking CD with polymeric networks, forming highly swellable drug-loaded nanogels.	Beta-cyclodextrin, cyclodextrin nanosponge	Crosslinkers: MBA, AMPS, and NVP; polymerization initiated by AIBN, KPS, and UV-radiation; particle size: 50–400 nm	[14,18,54,79]
Emulsification–solvent Evaporation	Applied for nano- and microgel preparation, allowing sustained drug release.	Gamma-cyclodextrin, hydroxypropyl-gamma-cyclodextrin	Emulsifier: PVA, Tween 80; droplet size control: ultrasonication; particle size: 100–500 nm	[48,75]
Crosslinking (chemical and physical methods)	Creates stable hydrogel matrices by polymerizing CD derivatives with crosslinkers.	Beta-cyclodextrin, carboxymethyl-beta-cyclodextrin	Chemical crosslinkers: glutaraldehyde, EPI, carbodiimide; physical methods: UV curing, freeze–thawing	[5,13,14,16,27,42,49,65]
pH-induced gelation	Utilized for forming pH-sensitive hydrogels that respond to acidic or basic environments.	Beta-cyclodextrin, hydroxypropyl-beta-cyclodextrin	pH-triggered drug release: swelling at pH 5–7; transition from sol to gel state at specific pH	[4,21,54,74]
Thermoresponsive gelation	Forms temperature-sensitive hydrogels that transition between liquid and gel states at physiological temperatures.	Hydroxypropyl-beta-cyclodextrin, randomly methylated beta-cyclodextrin	Gelation temperature: 28–37 °C; polymer systems: Pluronic, poloxamer, HPMC, PEO	[40,41,69,72]
Microneedle array technology	Used for transdermal drug delivery, allowing controlled drug release via microneedle patches.	Beta-cyclodextrin, hydroxypropyl-beta-cyclodextrin	Microneedle height: 300–600 µm; drug loading efficiency: 50–90%; patch swelling for controlled release	[42,76,77]

**Table 4:** Key factors affecting drug release in CD–hydrogel systems.

Key Factor	Effect on Drug Release	Common Cyclodextrins Involved	Key Technical Considerations	References
Cyclodextrin type and inclusion complex formation	Controls drug solubility and bioavailability, impacts release kinetics.	Beta-cyclodextrin, hydroxypropyl-beta-cyclodextrin, sulfobutyl ether-beta-cyclodextrin	Inclusion complex with molar ratio of 1:1 or higher, confirmed via FTIR, DSC, and PXRD	[1,3,4,7,13,16,19,22,43,61,68]
Hydrogel polymer composition and crosslinking density	Determines matrix porosity and drug diffusion rate, influences swelling capacity.	Beta-cyclodextrin-grafted polymers, carboxymethyl-beta-cyclodextrin, cyclodextrin nanosponges	Crosslinker concentration (0.1–2%) impacts gel stiffness, higher crosslinking → slower release	[27,40,51,65,77,80]
pH sensitivity of hydrogel	Regulates drug release in response to pH changes; important for oral and vaginal delivery.	Beta-cyclodextrin, hydroxypropyl-beta-cyclodextrin	pH-responsive swelling at pH 5–7 enhances release in physiological conditions	[4,21,80]
Temperature-sensitive gelation	Triggers drug release at physiological temperatures; suitable for ocular and transdermal applications.	Hydroxypropyl-beta-cyclodextrin, randomly methylated beta-cyclodextrin	Gelation occurs at 28–37 °C, modulated by Pluronic, poloxamer, and HPMC	[6,19,24,26,43]
Swelling and degradation of hydrogel matrix	Controls sustained release by gradual polymer erosion and swelling.	Cyclodextrin nanosponge, hydroxypropyl-gamma-cyclodextrin	Degradation time: hours to weeks, depending on hydrogel type	[14,20,23,42,56,73,79]
Drug hydrophobicity and molecular weight	Influences inclusion complex stability and diffusion rate in hydrogel.	Beta-cyclodextrin, hydroxypropyl-beta-cyclodextrin	Smaller hydrophobic drugs release faster, large molecules require hydrogel modifications	[20,40,60,79]
Ionic strength and osmotic effects	Affects gel structure and drug diffusion rate, particularly for mucoadhesive and ophthalmic gels.	Hydroxypropyl-beta-cyclodextrin, carboxymethyl-beta-cyclodextrin	High ionic strength delays release, osmotic balance crucial for in vivo stability	[30,32,58,78]
Microneedle patch swelling and penetration	Enhances transdermal drug diffusion, providing controlled long-term release.	Beta-cyclodextrin, hydroxypropyl-beta-cyclodextrin	Swelling time: 1–5 min, mechanical properties affect skin penetration	[42,76,77]
Emulsification and nanoparticle size	Smaller particle sizes enable faster diffusion, improving ocular and topical bioavailability.	Gamma-cyclodextrin, hydroxypropyl-gamma-cyclodextrin	Particle size: 100–500 nm enhances diffusion, emulsifiers stabilize nanogels	[8,19,48,75]
Release modulation via iontophoresis and electrostimulation	Enhances transdermal drug permeation, accelerates release in low-permeability drugs.	Hydroxypropyl-beta-cyclodextrin, beta-cyclodextrin	Electrical field: 0.1–1.0 mA/cm^2^ increases drug flux, used in pain management gels	[15,32,47,59]

**Table 5:** Drug Classes and Their CD-Based Formulations.

Drug or Therapeutic Agent	Examples of Drugs Used	Cyclodextrin (CD) Types Used	Key Features and Applications	References
Non-Steroidal Anti-Inflammatory Drugs (NSAIDs)	Ibuprofen, flurbiprofen, piroxicam	Beta-cyclodextrin, gamma-cyclodextrin, hydroxypropyl-beta-cyclodextrin	Used in topical, ophthalmic, and transdermal gels for pain relief and inflammation	[1,5,15,44,59,64,75,78]
Antifungal agents	Fluconazole, voriconazole, ketoconazole, clotrimazole, econazole	Sulfobutyl ether-beta-cyclodextrin, hydroxypropyl-beta-cyclodextrin, beta-cyclodextrin	Applied in mucoadhesive, thermosensitive, and ion-sensitive gels for vaginal, ocular, and systemic fungal infections	[2,3,6,7,19,50,53]
Ophthalmic drugs	Ciprofloxacin, dexamethasone, azithromycin, flurbiprofen, mitomycin	Alpha-cyclodextrin, hydroxypropyl-gamma-cyclodextrin, hydroxypropyl-beta-cyclodextrin	In situ gelling hydrogels used for sustained ocular release and improved bioavailability	[4,22,48,49,64,70,72]
Anticancer drugs	Curcumin, paclitaxel, 5-fluorouracil	Cyclodextrin nanosponge, randomly methylated beta-cyclodextrin, hydroxypropyl-beta-cyclodextrin	Applied in pH-sensitive, biodegradable, and thermoresponsive hydrogels for targeted chemotherapy	[17,21,27,35,56,65,71,77]
Antibiotics	Vancomycin, ciprofloxacin, azithromycin	Hydroxypropyl-beta-cyclodextrin, alpha-cyclodextrin, beta-cyclodextrin	Used in mucoadhesive and sustained-release hydrogels for periodontal, bone, and systemic infections	[22,43,46]
Antiviral drugs	Acyclovir, valacyclovir, cabotegravir	Beta-cyclodextrin grafted polymer, gamma-cyclodextrin, hydroxypropyl-beta-cyclodextrin	Studied in pH-responsive and transdermal hydrogels for herpes, HIV, and viral infections	[34,54,61,80]
Antipsychotic and neurological drugs	Clonazepam, asenapine, risperidone, armodafinil, paliperidone	Randomly methylated beta-cyclodextrin, hydroxypropyl-beta-cyclodextrin, hydroxypropyl-gamma-cyclodextrin	Used in nasal, buccal, and transdermal delivery for schizophrenia, epilepsy, and cognitive disorders	[26,28,31,45,74]
Cardiovascular and cholesterol-lowering Agents	Rosuvastatin, lovastatin, telmisartan	Beta-cyclodextrin polymeric nanogel, beta-cyclodextrin grafted hydrogel, beta-cyclodextrin microneedles	Applied in nanogel-based and mucoadhesive hydrogels for cholesterol reduction and blood pressure control	[14,76,79]
Hormone replacement and endocrine therapy	Insulin, estradiol, dehydroepiandrosterone	Methyl-beta-cyclodextrin, methyl-beta-cyclodextrin, hydroxypropyl-gamma-cyclodextrin	Used in vaginal and nasal gels for menopausal hormone therapy and insulin delivery	[30,33,38,67]
Antioxidants and natural therapeutics	Curcumin, rutin, resveratrol, gallic acid, babchi oil (Psoralea corylifolia), polyglycerol, peppermint	Cyclodextrin nanosponge, hydroxypropyl-beta-cyclodextrin, cyclodextrin nanosponge	Studied in topical and systemic hydrogels to enhance bioavailability and therapeutic efficacy	[20,27,40,65,66,81]

**Table 6:** Advancements and innovations in CD–hydrogel drug delivery.

Category	Key Observations and Insights	References
Synergistic cyclodextrin use	- Co-utilization of multiple cyclodextrin derivatives (e.g., HP-β-CD and γ-CD) enhances solubility, bioavailability, and retention in formulations for voriconazole, curcumin, dehydroepiandrosterone, and dexamethasone.	[6,17,32,38]
	- Sulfobutyl ether-beta-cyclodextrin (SBE-β-CD) reduces ocular irritation while improving drug permeation and retention in ion-sensitive in situ gels.	[7]
	- Gamma-CD metal–organic frameworks (γ-CD-MOFs) increase stability, photostability, and targeted hydrophobic drug delivery (e.g., curcumin, anticancer agents).	[17,27]
Hybrid polymers and smart architectures	- Hybrid formulations combining natural (e.g., chitosan) and synthetic polymers (e.g., Pluronic F127, PEG) improve gelation, mechanical strength, and sustained release kinetics.	[6,27,43,51]
	- Multi-responsive hydrogels (pH- and temperature-sensitive) facilitate multi-drug delivery, particularly in oncology and gastrointestinal treatments.	[14,22,43,56,67]
Next-generation drug release control	- Stimuli-responsive hydrogels allow spatiotemporal drug release control, crucial for targeted cancer therapies and mucosal drug delivery.	[43,56,67,80]
	- Ion-sensitive hydrogels enable precise ocular drug release, leveraging ionic fluctuations for on-demand administration.	[3]
Advanced encapsulation strategies	- Cyclodextrin-based nanosponges and nanogels enhance stability, bioadhesion, and sustained release (e.g., curcumin nanogels).	[17,27,65]
	- Gamma-CD metal–organic frameworks (MOFs) improve photostability and targeted drug localization, ideal for topical and anticancer applications.	[17,80]
Multifunctional and bioactive properties	- Cyclodextrin hydrogels offer dual antimicrobial and antioxidant properties, suitable for wound healing, tissue repair, and infection prevention.	[20,51,53,66]
	- Co-delivery of synergistic actives (e.g., curcumin and resveratrol) enhances cytotoxic efficacy and tissue penetration.	[27,65]
Enhanced patient compliance and non-invasive therapies	- Microneedle-integrated hydrogels enable painless, transdermal drug delivery (e.g., sildenafil citrate for erectile dysfunction and curcumin for treating melanoma).	[42,77]
	- Sustained-release formulations (7+ day drug profiles) improve compliance for chronic diseases (e.g., diabetes, schizophrenia).	[45,56,59,67]
Scalability and sustainable production	- Spray-drying, freeze-drying, and emulsification techniques facilitate scalable hydrogel production with high reproducibility.	[6,17,33,68]
	- Eco-friendly hydrogel development: green chemistry approaches, solvent-free processing, and sustainable crosslinkers align with regulatory trends.	[5,21,51]
Regenerative medicine and tissue engineering	- Cyclodextrin hydrogels serve as bioactive scaffolds for wound healing, tissue regeneration, and drug-eluting implants.	[13,21,77]
	- Integration of growth factors, peptides, and antimicrobial agents enhances regenerative capacity, particularly for inflammatory skin disorders, burns, chronic wounds, and post-surgical tumoral recovery.	[13,21,66]
Comparative performance vs. commercial formulations	- Cyclodextrin-based hydrogels consistently outperform commercial counterparts, exhibiting superior drug solubility, permeability, and bioactivity.	[5,32,41,53]
	- Example: fluconazole hydrogels demonrate larger antimicrobial inhibition zones (30 mm vs. 24 mm) compared to commercial antifungal formulations.	[19,53]
Emerging frontiers in drug delivery	- Cutting-edge applications include gene therapy, HIV prevention, and neurodegenerative disease treatment (e.g., cabotegravir-loaded hydrogels for HIV prophylaxis).	[33,61,63]
	- Microneedle-assisted cyclodextrin systems are being explored for targeted dermatological treatments, including melanoma therapy (curcumin patches).	[77]
Optimized drug delivery for cancer	- Injectable hydrogels, pH-sensitive carriers, and CD-MOFs are transforming tumor-targeted therapy, reducing systemic toxicity while enhancing localized drug delivery.	[21,56]
	- Combination therapies in hydrogel matrices (e.g., chemotherapeutics + antioxidants) significantly improve therapeutic outcomes.	[17,65]

## Data Availability

No new data were created or analyzed in this study. Data sharing is not applicable to this article.
